# Poverty and disability in low- and middle-income countries: A systematic review

**DOI:** 10.1371/journal.pone.0189996

**Published:** 2017-12-21

**Authors:** Lena Morgon Banks, Hannah Kuper, Sarah Polack

**Affiliations:** International Centre for Evidence in Disability, Department of Clinical Research, London School of Hygiene & Tropical Medicine, London, United Kingdom; TNO, NETHERLANDS

## Abstract

**Introduction:**

Disability and poverty are believed to operate in a cycle, with each reinforcing the other. While agreement on the existence of a link is strong, robust empirical evidence substantiating and describing this potential association is lacking. Consequently, a systematic review was undertaken to explore the relationship between disability and economic poverty, with a focus on the situation in low and middle income countries (LMICs).

**Methods:**

Ten electronic databases were searched to retrieve studies of any epidemiological design, published between 1990-March 2016 with data comparing the level of poverty between people with and without disabilities in LMICs (World Bank classifications). Poverty was defined using economic measures (e.g. assets, income), while disability included both broad assessments (e.g. self-reported functional or activity limitations) and specific impairments/disorders. Data extracted included: measures of association between disability and poverty, population characteristics and study characteristics. Proportions of studies finding positive, negative, null or mixed associations between poverty and disability were then disaggregated by population and study characteristics.

**Results:**

From the 15,500 records retrieved and screened, 150 studies were included in the final sample. Almost half of included studies were conducted in China, India or Brazil (n = 70, 47%). Most studies were cross-sectional in design (n = 124, 83%), focussed on specific impairment types (n = 115, 77%) and used income as the measure for economic poverty (n = 82, 55%). 122 studies (81%) found evidence of a positive association between disability and a poverty marker. This relationship persisted when results were disaggregated by gender, measure of poverty used and impairment types. By country income group at the time of data collection, the proportion of country-level analyses with a positive association increased with the rising income level, with 59% of low-income, 67% of lower-middle and 72% of upper-middle income countries finding a positive relationship. By age group, the proportion of studies reporting a positive association between disability and poverty was lowest for older adults and highest for working-age adults (69% vs. 86%).

**Conclusions:**

There is strong evidence for a link between disability and poverty in LMICs and an urgent need for further research and programmatic/policy action to break the cycle.

## Introduction

Globally, it is estimated that 15% of the global population–representing 1 billion people–is living with a disability [1].

Poverty and disability are believed to operate in a cycle, with the one reinforcing the other. In low- and middle-income countries (LMICs) in particular, conditions associated with poverty, such as lack of access to healthcare, inadequate water and sanitation, malnutrition and poor living conditions, increase the risk of disability [2, 3]. Even in the absence of absolute poverty, social inequalities and relative poverty can lead to stress and social exclusion, which can worsen both mental and physical health and functioning [4]. On the other side, disability can lead to exclusion from work, education and healthcare, as well as high healthcare and other expenses, which can cause or exacerbate poverty [1, 5–7].

While there is broad agreement of a link between disability and poverty, the empirical evidence for this association is less clear. Typically, a small set of statistics are routinely cited–for example, that people with disabilities are twice as likely as people without disabilities to be living in poverty [3, 8]. However, despite their widespread citation, upon tracing to the original source, many such figures were found to be based on decidedly weak evidence [8].

A key focus of the development agenda, including the 2030 Sustainable Development Goals (SDGs), is the alleviation of poverty in all its forms [9]. The failure to include disability issues in the predecessor Millennium Development Goals has been recognised as leading to the exclusion of people with disabilities from its benefits, potentially widening inequalities between people with and without disabilities [10]. Consequently, the SDGs have striven to ensure “no one is left behind” by promoting a stronger focus on disability, including in the call to disaggregate data monitoring progress by disability status.

While the interplay of poverty and disability is increasingly identified as a major limitation to growth and development, the lack of robust empirical evidence to inform policy and programmatic decisions may impede effective action. Efforts to provide a more cohesive understanding of the association between disability and poverty have highlighted a need for further research in this field to both substantiate and describe linkages. A critical review on poverty, health and disability in LMICs conducted in 2011, concludes that while some studies do show strong links, the evidence base is relatively limited and the relationship between poverty, disability and health may be more complex than previously assumed. As acknowledged by the authors, however, this was a non-systematic review which identified a relatively small collection of studies [8]. Similarly, a review on childhood disability and home socio-economic circumstances in LMICs found that quantitative evidence of an association was inconclusive and inconsistent [11]. Both of these reviews used only general terms for disability in their search strategies (e.g. “disability”, “handicap”) and did not include terms for specific disability types (e.g. vision impairment, intellectual impairments) and thus may have potentially excluded many relevant studies.

While poverty can take many forms, economic measures (e.g. income, assets, consumption) are the most frequently used in international comparisons and provide valuable information about an individual or household’s well-being, relative or absolute deprivation and ability to meet basic needs [8]. We have thus undertaken a systematic literature review of empirical studies that compare the level of economic poverty between people with and without disabilities in LMICs. By using systematic methods and extensive search strategies, this review aims to provide a more comprehensive analysis which will build on the existing efforts.

## Methods

This systematic review explores the relationship between disability and poverty, including whether characteristics such as impairment type, gender or study location modify this relationship. The review was conducted in line with PRISMA guidelines (S1 Table for PRISMA Checklist) [12].

### Search strategy

The following ten electronic databases were searched in March 2016 for studies assessing the relationship between disability and economic poverty: EMBASE, PubMed, MEDLINE, Global Health, Web of Knowledge, Academic Search Complete, FRANCIS, ERIC, Social Policy & Practice and EconLit. Additionally, references of relevant review articles were checked to identify additional potential studies.

Comprehensive search terms for poverty, disability and low and middle income countries (LMICs) were identified through MeSH/Emtree as well as from those used for systematic reviews on similar topics (see S1 File for sample search string) [13, 14]. The search was limited to English-language titles and articles published between 1990- March 2016.

### Inclusion/exclusion criteria

Since the purpose of this review focused on the published evidence for a relationship between poverty and disability in LMICs, only papers involving all three of these topics were included. Papers exploring both directions of association between poverty and disability, as well as those in which the directionality was not evident, were included in the final sample. We included studies that assessed disability broadly (e.g. through self-reported functional or activity limitations) as well as studies that focussed on specific impairments or disorders (vision, hearing and physical impairments, intellectual disability and mental disorders) measured using standardised tools or clinical measures. Poverty was restricted to economic measures, namely income, expenditures, assets and/or socioeconomic status (SES). SES measures could include a range of indicators as part of their composition (e.g. housing characteristics, access to services, education level); however to be eligible for inclusion, measures of SES had to include at least one economic indicator (income, expenditures, or assets) [15]. Poverty could be defined as absolute or relative.

Studies with an epidemiological design were eligible for inclusion. Only studies with comparison groups (i.e. to allow comparison of people with disabilities to people without disabilities) were included. Qualitative studies, review articles and case reports were excluded.

### Study selection

Articles were screened by one reviewer (LMB) first by titles, then abstract and then finally by full paper to determine eligibility. Ten percent of the abstracts were dually reviewed by a second reviewer (SP or HK) to check for agreement.

The full-text of all eligible studies were assessed against quality criteria [14] independently by two reviewers (LMB with either HK or SP; see Table 1 for the quality assessment criteria). Differences between the reviewers were discussed and a consensus was reached on all papers. We excluded studies deemed to have a high risk of bias.

**Table 1:** Quality assessment criteria and ratings.

**Assessment criteria by study design** *All study designs* • Study design, sampling method is appropriate to the study question • Adequate sample size, e.g. sample size calculations undertaken • Response rate reported and acceptable (>70%) • Disability/impairment measure is clearly defined and reliable • Economic measure is clearly defined and reliable • Potential confounders taken into account in analysis • Confidence intervals are presented *Case control (additional criteria)* • Cases and controls are comparable • Cases and controls are clearly defined *Cohort (additional criteria)* • Groups being studied are comparable at baseline • Losses to follow up are presented and acceptable	
**Risk of bias**:	
Low	All or almost of the above criteria were fulfilled, and those that were not fulfilled were thought unlikely to alter the conclusions of the study
Medium	Some of the above criteria were fulfilled, and those not fulfilled were thought unlikely to alter the conclusions of the study
High	Few or no criteria were fulfilled, and the conclusions of the study were thought likely or very likely to alter with their inclusion. These studies were excluded from the final sample

Adapted from Lund et al, 2010 [14]

### Data extraction and analysis

Data extracted from the final selection of articles included:

- Study Design
- Method of assessment (poverty and disability),
- Setting (country, site of recruitment),
- Population characteristics (disability type, gender and age)
- Primary research outcome (measure of association between disability and poverty: univariate and multivariate).

In addition, although terms for employment were not included in the search strategy, the association between disability and employment status was recorded as a secondary outcome measure for the studies that conducted these analyses. All extracted values were checked by the second reviewer (SP or HK) to ensure accuracy.

In classifying study outcomes, an association was classified as ‘positive’ if either: a) the disability measured was significantly more common among poorer compared to wealthier economic groups or b) people with disabilities were significantly poorer compared to people without disabilities. Reverse associations (e.g. disability was significantly less common among poorer compared to wealthier economic groups) were categorised as ‘negative’. All classifications of association were made based on statistical significance, after adjusting for confounding (for studies employing multivariate analyses). Consequently, if findings did not achieve statistical significance after adjustment for at least one measure of the relationship between disability and poverty, they were labelled as having ‘null association’. If studies presented more than one measurement of association, it was classified as positive or negative if at least one association was statistically significant and the others were null; if both positive and negative statistically significant associations were found, the study was classified as ‘mixed’.

Proportions of studies finding positive, negative, null or mixed associations were then disaggregated by study characteristics, including disability/impairment type, age group of the sample (children, adults, older adults) and poverty indicator used, to explore whether such characteristics modify any relationship between disability and poverty.

## Results

**Fig 1:**
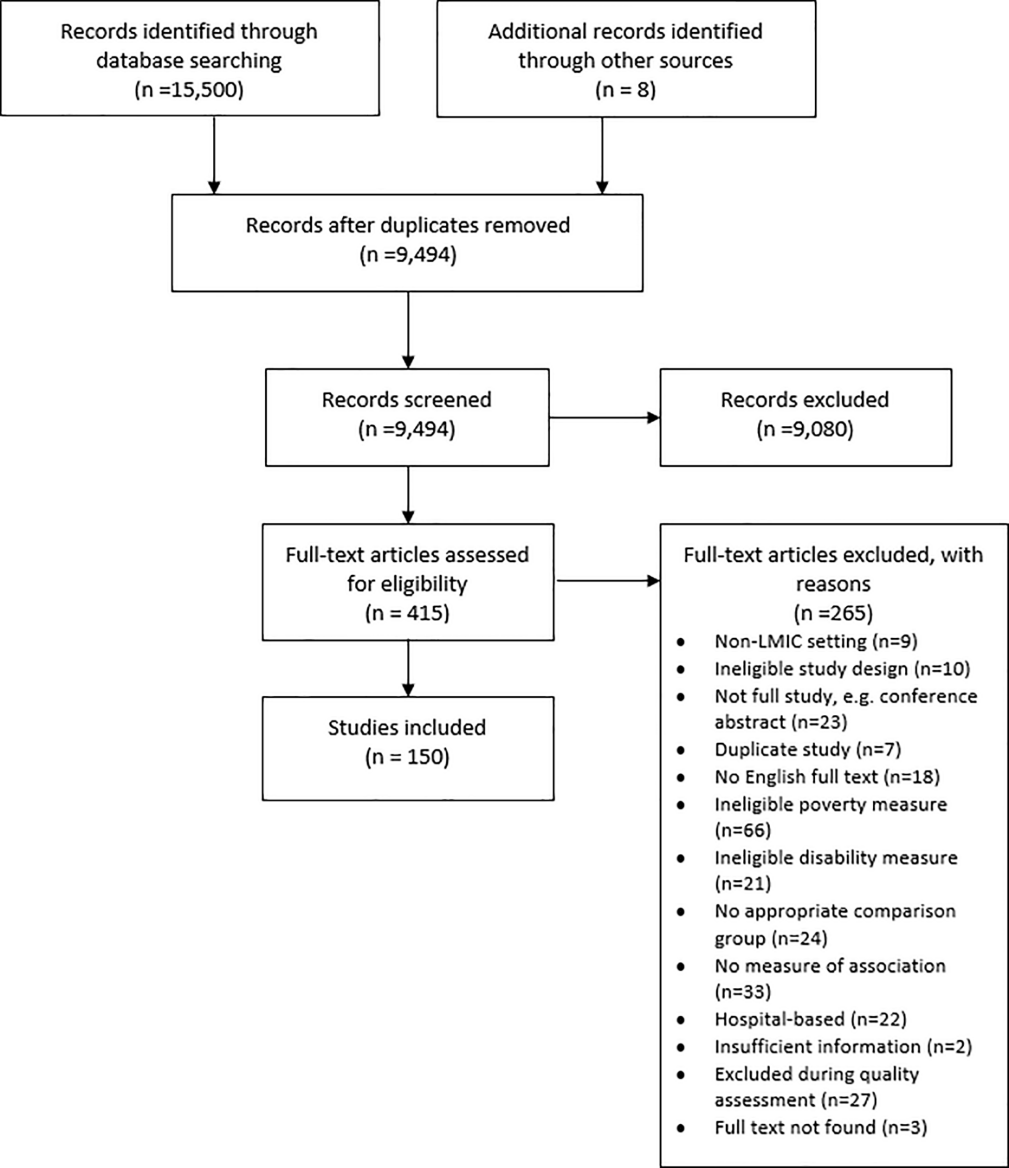
Flowchart of search results.

The database search generated a total of 15,500 records (9,494 after duplicates removed and years restricted), of which 7,534 and 1,546 records were excluded in the title and abstract screening, respectively. The full-texts of 415 articles were then assessed for inclusion. Of these 265 were deemed ineligible and 3 untraceable. A further 27 articles were excluded during the quality assessment. An additional 8 eligible articles were identified from reference lists of included articles and other reviews, providing a final sample of 150 studies (Fig 1)(see S2 File for included study citations).

### Overview of study characteristics

**Fig 2:**
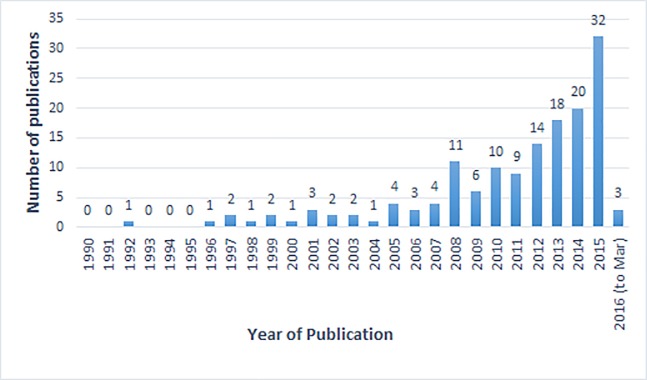
Number of included studies by year of publication.

Table 2 shows a breakdown of study characteristics. The majority of the included studies (almost 90%) were published from the mid-2000s onwards (Fig 2: Number of included studies by year of publication). Geographically, the largest number of studies were conducted in East Asia & the Pacific (n = 39, 27%; China = 29) followed by Latin America & the Caribbean (n = 31, 19%; Brazil n = 26), South Asia (n = 26, 20%; India n = 17), Sub-Saharan Africa (n = 22, 15%), Middle East/North Africa (n = 11, 8%) and Europe/Central Asia (n = 4, 3%). Of note, almost half of included studies were conducted in China, India or Brazil (n = 70, 48%). In addition, 16 studies were multi-regional. By country income group at time of data collection [16], study settings were relatively evenly split (low-income, n = 38; lower-middle, n = 42; upper-middle, n = 48). (See S2 Table for summarised extraction table)

**Table 2:** Characteristics of included studies.

	* *	Number^a^	%
***Region***	East Asia/Pacific	40	27
	Latin America/Caribbean	31	21
	South Asia	26	17
	Sub-Saharan Africa	22	15
	Middle East/North Africa	11	7
	Europe/Central Asia	4	3
	Multi-region	16	11
***Disability type***^b^	Visual impairment	12	8
	Hearing impairment	2	1
	Physical impairment	12	8
	Intellectual/cognitive impairment	23	15
	Mental disorders	73	49
	Mixed impairments/functional limitations	37	25
***Disability measure***	Impairment Activity/functional limitations Mixed	114 32 4	76 21 3
***Location***	Rural	17	11
	Urban	50	33
	Both	83	55
***Study design***	Cross-sectional	124	83
	Case-control	11	7
	Cohort	13	9
	Other	2	1
***Setting***	Community-based	133	89
	Hospital/clinic-based	6	4
	Schools	9	6
	Other	2	1
***Sample size***^a^	Smallest	85	
	First quartile (25^th^ percentile)	1,188	
	Median (50^th^ percentile)	3,591	
	Third quartile (75^th^ percentile)	10,667	
	Largest	2,600,000	
***Age group***^c^	Children	23	15
	Adults	41	27
	Older adults	48	33
	Mixed ages	37	25
***Income group***	Low	38	25
	Lower-middle	42	28
	Upper-middle	48	32
	Mix	16	11
***Poverty indicator***^b^	Income	82	55
	SES	36	24
	Assets	30	20
	PCE	10	7
	Other	2	1
***Risk of bias***	Low	81	54
	Medium	69	46

^a^ Except in the case of sample size, where the number of participants is given

^b^ Percentages totals more than 100% as some studies examined more than one category.

^c^ Age group cut-offs varied based on author and country-specific designations but typically children <18 years, adults 18–60 years, older adults 60+ years.

Concerning study design characteristics, the vast majority (n = 124, 83%) were cross-sectional studies. The remainder were comprised of 11 case-control, 13 cohort studies, one pre-post and one ecological study. The majority of studies recruited participants from the general population (n = 133, 89%), while hospitals/clinics (n = 6), and schools (n = 9) were utilized for the rest. In terms of the study population age groups, 48 studies focused on older adults only (33%), 41 included working age adults only (27%), 23 included children/adolescents only (15%) and 37 included participants across age categories (25%).

The majority of studies (n = 114, 77%) focussed on specific impairment types (e.g. vision or hearing impairment) and most used clinical examinations or standardised, objective assessment tools. However, some studies (n = 33, 23%) used indicators such as self-reported activity or functional limitations that are more in line with the World Health Organisation International Classification of Functioning, Disability and Health model of disability [17]. Mental disorders (n = 73, 49%) were the most frequently assessed disability type, followed by intellectual/cognitive impairments (n = 23, 15%), functional limitations/mixed impairment types (n = 37, 25%), sensory impairments (n = 14, 9%) and physical impairments (n = 12, 8%).

Income was the most frequently measured indicator for economic poverty (n = 82, 55%). Most studies reported total or per capita family/household income, while a small number reported individual or household head income, satisfaction with income and change in income over the life course. SES was the second most common economic measure (n = 36, 24%), followed by asset ownership (n = 30, 20%). The majority of SES indices were based on ownership of assets and household characteristics while some included other more multidimensional facets such as education, occupation, income, sanitation facilitates and use of services. A smaller number of studies collected data on per capita expenditure (n = 10, 7%).

#### Risk of bias in included studies

Of the included studies, 54% were deemed to have a low risk of bias and 46% were medium; a further 27 studies were excluded from this review as they were deemed to have a high risk of bias that was likely to alter their findings related to the relationship between disability and economic poverty.

Major sources of bias across studies included the lack of clearly defined, valid economic and/or disability measures. For disability measures, several studies measured disability through self-report of impairments or clinical diagnoses, or through a binary question on whether the respondent identified as disabled; both of these approaches are considered to underestimate the prevalence of disability, skewing estimates to more severe or “visible” forms of disability [18–20]. For economic measures, some metrics were inadequate to detect finer differences between populations that were mostly poor [21] or lacked sufficient validation.

Lack of adequate adjustment for confounding was also a concern, as, 20 studies (13%) were bivariate analyses only. Finally, low response rates and non-population based samples, were also sources of bias.

### Association between disability and poverty

Overall, the vast majority of studies (n = 122, 81%) found evidence for a positive relationship between disability and poverty. The remainder was comprised of 23 studies (16%) that found no significant association, three (2%) that found a negative relationship and two with mixed findings. The study findings are disaggregated by study characteristics in Table 3.

**Table 3:** Association of poverty and poverty by study characteristics.

		Association of poverty with disability				
		*Positive*	*Null*	*Negative*	*Mixed*	*Total*
		%	%	%	%	N
**Overall**		81%	16%	2%	1%	150
**Disability/ impairment type**^a^	Sensory impairment	78%	17%	0%	6%	18
	Physical impairment	80%	15%	5%	0%	20
	Intellectual/cognitive impairment	69%	31%	0%	0%	26
	Mental disorders	87%	11%	3%	0%	75
	General disability/functional limitations	80%	14%	3%	3%	35
**Disability measure**^a^	Impairments	81%	16%	3%	1%	115
	Activity/functional limitations	79%	19%	0%	3%	33
	Mix	100%	0%	0%	0%	3
**Age group**^a^	Children	78%	15%	0%	7%	27
	Adults	86%	12%	2%	0%	42
	Older adults	69%	27%	4%	0%	49
	Mixed ages	92%	8%	0%	0%	36
**Poverty indicator**^a^	Income	83%	16%	0%	1%	82
	SES	81%	14%	3%	3%	36
	Assets	77%	20%	3%	0%	30
	PCE	60%	30%	10%	0%	10
**Region**^a^	Latin America/Caribbean	60%	30%	10%	0%	60
	East Asia/Pacific	80%	17%	2%	2%	60
	Sub-Saharan Africa	51%	44%	4%	0%	68
	South Asia	79%	19%	2%	0%	42
	Middle East/North Africa	87%	7%	7%	0%	15
	Europe/Central Asia	36%	64%	0%	0%	14
	Multi-region	100%	0%	0%	0%	8
**Income group**^a^	Low	59%	4%	3%	1%	95
	Lower-middle	67%	28%	5%	0%	100
	Upper-middle	72%	26%	1%	0%	69
**Gender**^a^	Female	87%	14%	0%	0%	30
	Male	86%	14%	0%	0%	22
**Setting**	Rural	82%	18%	0%	0%	17
	Urban	82%	20%	2%	0%	50
	Both	82%	12%	2%	2%	83
**Risk of bias**	Low	88%	10%	1%	1%	81
	Medium	74%	22%	3%	1%	69

^a^ Findings within studies have been disaggregated, where possible

#### Disaggregation by disability/impairment types

The relationship between disability and poverty was apparent across all types of impairments/disability.

Of the 75 papers that focussed on mental disorder, 87% found evidence of a positive relationship with poverty. Papers in this category could be subdivided into depression/anxiety (n = 31), common mental disorders [22] (n = 12) and other (n = 32). For depression, 26 papers found a positive association with poverty, a null association and one study found a negative association between lifetime prevalence of depression and assets in older adults in Nigeria, though the analysis was unadjusted by potential confounders. The relationship between common mental disorders and poverty was positive for ten studies, and null for the remaining two studies. For other mental disorders, 29 found a positive association, two found no association and one study found a negative relationship between per capita expenditure and psychiatric disorders.

Eighteen studies included analyses on sensory impairments, with 12 focusing on visual impairment, two on hearing impairment and three on both. Of these, 14 of 18 studies (78%) found a positive association with poverty. Additionally, three studies found no significant association between visual impairment and poverty; two of these studies performed unadjusted analyses only. One study in Vietnam reported mixed findings, with a positive association between hearing impairment and poverty, but a negative association with visual impairment.

Eighteen of the included studies evaluated the link between poverty and physical impairment. Fourteen of these studies (78%) found evidence of a positive association. The remaining four studies found no significant difference in poverty level between people with and without a physical impairment.

Among the 35 studies with more global measures of disability (e.g. mixed impairment types, functional limitations), 28 (80%) found a positive association with poverty and five studies found no significant difference in poverty between people with and without disabilities. Two studies reported mixed findings and one found a negative relationship.

There were 26 studies which reported on the association between poverty and intellectual/cognitive impairments, of which 69% found evidence of a positive relationship. Most studies in this category (n = 16) focused on dementia and cognitive impairment in older adults. Of these, eight (50%) showed a null association. The other ten studies in this category (all but two of which were conducted in children), all found a positive association.

Eighty-nine studies disaggregated data by either levels of poverty or severity of disability. Of these, most (61 of 89, 69%) found the strength of the association between disability and poverty increased with increasing level of poverty/severity of disability. Four studies with negative associations also found a dose response relationship.

Finally, there was little difference between studies that used impairment-based measures of disability (93 of 114, 81%) compared to those that focused on activity or functional limitations (25 of 33, 79%).

#### Disaggregation by study setting region and country income group

By region, studies set in the Middle East & North Africa and East Asia & the Pacific countries were most likely to find a positive relationship between disability and poverty, with respectively 87% and 80% of analyses finding significant associations. In contrast, studies from Sub-Saharan Africa and Europe & Central Asia were least likely to report positive associations, with only 51% and 36% of analyses finding positive associations.

By country income group at the time of data collection, the proportion of country-level analyses with a positive association increased with the rising income level, with 59% of low-income, 67% of lower-middle and 72% of upper-middle income countries finding a positive relationship.

#### Disaggregation by other factors

By age group, the proportion of studies reporting a positive association between disability and poverty was lowest for older adults and highest for working-age adults (69% vs. 86%). Studies with mixed age groups–which comprised predominantly working-age adults–were mainly positive (92%).

The positive relationship between disability and poverty was consistent by economic indicator, though it was the least consistent for the nine studies using per capita expenditure as the measure (60% positive).

The majority of studies’ settings included both rural and urban areas (n = 83). For studies limited to either or rural or urban settings, there was little difference in their findings on disability and poverty.

By risk of bias, studies with an assessed low risk were slightly more likely to find a positive association between disability and economic poverty (88% vs 74% for studies with a medium risk of bias).

Finally, while the majority of studies did not disaggregate by gender, for the 30 which did provide separate analyses for men and women (22 disaggregated studies, 8 studies among women only), the relationship between disability and poverty did not differ between men and women (86% vs 87% for men and women respectively).

#### Evidence on directionality of association

As 83% of the included studies are cross-sectional, it is difficult to ascertain the directionality of the association between disability and poverty in their analyses. The thirteen cohort studies and one pre-post study however, provide some indication. In all these studies, the focus was on how economic poverty impacted the risk of developing disability and all but one found that lower financial status was associated with an increased risk of developing a disability. Nine studies focused on development of mental disorders among different economic groups, with all but one finding a positive association. Additionally, three studies found a positive link between lower household income and developmental delay in children. Two studies on older adults reported individuals from poorer backgrounds were more prone to functional decline and dementia than their wealthier peers.

No longitudinal studies were identified that explored whether disability could lead to poverty.

#### Association between disability and employment status

While this review did not systematically explore the relationship between disability and employment, we did extract data from included studies as a scoping exercise to understand potential drivers of the relationship between disability and poverty.

In total, 35 of the studies included in this review assessed the relationship between disability and employment. Of these, 26 (74%) found a positive association (i.e. disability was significantly more common among non-employed versus employed groups or people with disabilities were significantly more likely to be non-employed compared to people without disabilities). The remaining eight studies found no significant association between employment status and disability, with one finding a negative association.

## Discussion

This systematic review finds strong evidence to support the link between disability and economic poverty, with 122 of 150 (81%) included studies reporting a statistically significant, positive relationship between these two variables. This large and comprehensive review therefore provides a robust empirical corroboration to the more theoretical arguments of a link between disability and economic poverty.

In addition to the large proportion of studies reporting a positive association between disability and economic poverty, other factors in line with the Bradford Hill criteria further substantiate the plausibility of a genuine link [23]. First, the observed relationship remained significant after authors adjusted for a range of confounders, such as age, gender, area of residence and level of education. Second, the trend of association was mostly consistent across regions, impairment types, study designs and age groups. Third, in the studies which disaggregated data by either levels of poverty or severity of disability, most (61 of 89, 69%) found evidence of dose response: namely, the strength of the association between disability and economic poverty increased with increasing level of poverty/severity of disability. Additionally, as explained through the disability-poverty cycle [2] and social determinants of health inequalities [4, 24], there are plausible mechanisms to explain how disability could contribute to economic poverty and vice versa.

Only five studies found a significant negative association (two of which were mixed) between disability and economic poverty [25–29], and these can be at least partially explained by mitigating factors. First, Pham et al found a significant negative relationship between visual impairment in children and household income, even though analyses of other impairment types in the study showed a positive association [25]. The finding was explained by the authors as likely resulting from additional schooling in wealthier households, with eyestrain from increased engagement in activities such as reading or using a computer heightening the risk of visual impairment. Second, Kuper et al. reported mixed findings on the association between disability and asset ownership in a multi-country study of children who were part of the Plan International Child Sponsorship Programme [26]. As criteria for entering into the programme is based on poverty and other forms of vulnerability, the comparator group of children without disabilities may have other characteristics (e.g. ethnic/religious/racial minority, orphans), which may be greater drivers of poverty compared to disability in certain contexts.

Third, Nakua et al. found arthritis/joint pain was more common in higher SES groups in Ghana; however this findings is likely explained by the measure of disability, which was self-report of a clinical diagnosis [27]. As poverty and poorer access to healthcare are linked [30], the observed association may be more reflective of the relationship between wealth and receiving needed medical attention. Fourth, Islam et al report an increase in psychiatric disorders with rising per capita household expenditures in Bangladesh [28]; the authors attributed this finding as potentially due to less familiarity and comfort with interview schedules used to ascertain psychiatric disorders among lower individuals from lower SES groups. Finally, Gureje et al. found a negative association between depression and asset ownership [29]; however, the analysis did not control for any potential confounders.

Twenty-three studies found no significant association between disability and economic poverty. However, eighteen of these studies found evidence of a positive relationship with other broader indicators of poverty (e.g. education, malnutrition, employment) not covered in this review [5, 31–47], indicating the value of more multi-dimensional approaches to studying poverty.

While overall the relationship between disability and economic poverty was consistent when disaggregated by a range of study characteristics, some variations were observed. For example, studies set in low-income countries or in certain regions (notably sub-Saharan Africa and Europe/Central Asia) were less likely to observe a relationship between disability and poverty. Some of this variation may be due to challenges accurately and appropriately measuring poverty in complex and varying economies. For example, in settings with high absolute poverty, differentiating between households or individuals may be challenging and the studies may have been under-powered to detect these small differences. Furthermore, accurately capturing true economic well-being in economies defined by the dominance of the informal sector, non-cash remunerated work, irregular flows of income and complex community-based resource sharing arrangements requires careful methodological consideration [48]. An alternative explanation for these trends is that people with disabilities are “left behind” as regions develop economically, so that the gap in poverty between those with and without disabilities will be larger in areas that are less poor.

Similarly, the strength of the relationship between disability and economic poverty differed slightly by age group. Analyses focused on older adults were slightly less likely to be positive (69%), compared to working-age adults (86%) and children (78%). In particular, dementia and cognitive impairment in older adults was not highly correlated with economic poverty (8 of 16 studies finding a positive association). If onset of disability occurs later in life, these individuals may have established more safeguards to protect against sliding into poverty than individuals who develop disabilities earlier life and face exclusion throughout the life course. Additionally, as economic poverty has been linked consistently to lower life-expectancy [24], poorer individuals who survive into older age may be healthier than their wealthier counterparts.

While these findings provide clear evidence of correlation between disability and economic poverty, it is difficult in most cases to ascertain the direction of association given that 83% of the included studies are cross-sectional. Fourteen longitudinal studies [34, 49–59]—most of which focused on mental health conditions–assessed the risk of developing disability among different economic groups; all but one [34] found a positive association, providing evidence supporting the social determinants of health theory [4, 24]. The findings for mental health in particular are corroborated by studies in high income countries [60], which find the daily stresses associated with lower social and economic position, combined with lower access to healthcare and other services, can increase the risk of mental health conditions.

The high proportion of studies showing a positive relationship between disability and economic poverty observed in this review stands in contrast to other reviews [8, 11], where findings were more mixed. Several factors may explain this difference. The search strategy for this study which used terms for both general disability as well as specific impairments/conditions and used systematic searching across multiple databases led to the inclusion of substantially more studies than either of the other reviews, thus greatly broadening the pool from which to draw evidence. Additionally, as the others used multidimensional conceptualizations of poverty whereas this review focused solely on the economic component, the divergence in findings may simply underscore the difference in definitions.

### Limitations

There are some limitations that should be taken into account when interpreting the findings of this review. First, if studies showing a negative or null association were less likely to be published–resulting in publication bias–the association between economic poverty and disability could be overestimated. However, as most included papers were not focused explicitly on exploring the relationship between economic poverty and disability and instead either investigated this association as a secondary measure or as part of a multivariable analysis, it is unlikely that this source of potential bias was important. Second, we only focussed on economic definitions of poverty and did not include more multidimensional measures such as access to health, education or food security, which presents a limited view of poverty [61]. Third, as almost half of included studies were conducted in either Brazil, China or India, the findings of this review may be biased towards reflecting the conditions in those countries, which may differ from other LMICs. Similarly, other country-level factors that could affect the strength of the observed association–such as disability prevalence, availability and access to health and rehabilitation services, social protection and other supports–could not be included in the analysis as reliable, comparable data on these indicators are not available in most countries. Fourth, since the majority of included studies (n = 122, 83%) were cross-sectional, it was not possible to comment on the directionality of association in most cases, particularly of disability leading to decreased economic status. Fifth, the wide range of tools used to measure both disability and economic poverty–which varied in their sensitivity and validity–could affect the comparability and reliability of findings.

Finally, this review likely underestimates the full magnitude of the association between disability and economic poverty. Increasingly, experts are pointing to the need for an adjusted poverty line for people with disabilities to account for additional costs associated with disability incurred as a result of the need for assistive devices, personal supports, extra transport or higher medical/rehabilitation expenses [7, 62, 63]. As recognition of and methods for incorporating extra disability-related costs are underdeveloped, little evidence currently exists on relative poverty between people with and without disabilities taking into account this higher economic threshold needed to meet basic needs.

### Implications for future research

#### On the relationship between disability and economic poverty

While this review did identify a large number of studies exploring the relationship between disability and economic poverty, there is still need for further research in this area to understand how the relationship changes over time, place and between groups. To improve the quality of research in this area, there is a need for more standardised, robust measures of both disability and economic poverty to enable comparisons across contexts and over time. For example, a major source of bias in studies included in this review was the lack of detail on and reliability of economic poverty measures. This reinforces findings in Cooper et al’s review on measuring poverty in psychiatric epidemiology, which highlights the pressing need for more critical and systematic approaches to assessments of poverty in varying contexts [21].

Longitudinal studies are particularly needed, especially in measuring the economic impacts after the development of disability as no study identified focused on this direction of association. Furthermore, as both disability and economic poverty are dynamic and can fluctuate across the life-course, understanding the impact of these variations over time is also important.

#### Other forms of poverty

While economic poverty is a key metric for understanding and comparing well-being, deprivation and ability to meet basic needs, research exploring the relationship between disability and more multi-dimensional forms of poverty is also needed. By using a range of indicators–such as lack of education and engagement in decent work, inadequate living standards and poor health–multidimensional poverty may better capture the complexity of poverty and in turn assist in informing more nuanced strategies for poverty alleviation and disability prevention [61].

Furthermore, more research is needed on intra-household poverty. Most economic and many multidimensional measures of poverty use the household as the unit of analysis, which may obscure uneven distribution of resources or opportunities within the household [6]. For example, limited emerging research indicates that people with disabilities may fare worse compared to other household members on indicators such malnutrition and access to education [45, 64]–which could be indicative of unequal allocation of resources or additional barriers to meeting basic needs among people with disabilities. Furthermore, additional research is needed on the extra-costs of disability. In particular, gaps in the evidence include: (1) the overall magnitude and sources of these costs, (2) whether individuals are actually able to afford and access all needed goods and services, and (3) the impact of these expense on functioning as well as social and economic well-being [63].

#### “Causes of causes” and appropriate interventions

While this systematic review has provided clear evidence of a link between disability and economic poverty, further research is needed to understand what Marmot calls the “causes of causes” [65]: the underlying social, political and economic conditions that give rise to the link between disability and economic poverty. Access to health (including rehabilitation), education and employment may explain some of the relationship between disability and economic poverty, potentially in both directions. While this review identified that people with disabilities were more likely to not be working, since work status was a secondary measure without specific search terms, the observed association–as well as other potential drivers such as access to health and education–deserve further attention in separate systematic reviews. Understanding in greater depth how specific drivers impact the relationship between disability and economic poverty can help identify effective and appropriate interventions and strategies to break the cycle. To this end, attention will need to be given to how drivers vary among individuals and contexts, for example by gender, age and rural/urban settings.

Similarly, more research is needed to understand the impact of economic poverty on the lives of people with disabilities, as well as what existing interventions are effective at reducing poverty among people with disabilities. For example, exploring whether current poverty alleviation and social protection programmes are sufficiently disability-inclusive, as well as the impact of participation in both reducing disablement and/or decreasing poverty among people with disabilities is essential for policy and planning [66]. Similarly, given the finding in this review of a stronger association between disability and poverty as countries grow economically, it is critical to determine if and why people with disabilities are being “left behind” from the promise of economic growth and development.

## Conclusion

Failure to address the interaction between disability and poverty will undoubtedly stall economic growth and development, including in meeting the SDGs. With 81% of studies reporting a link between economic poverty and disability, the results of the systematic review provide a robust empirical basis to support the theorized disability-poverty cycle. Furthermore, as people with disabilities often incur additional expenses related to their disability (e.g. assistive devices, extra transportation) and thus may require a higher minimum threshold to meet basic needs [7], these findings likely underestimate the true extent of economic poverty among people with disabilities. Considering people with disabilities comprise upwards of 15% of the global population [1], neglecting to make poverty alleviation and development programmes disability-inclusive bars access to a substantial proportion of the population, significantly reducing their potential impact and enhancing inequalities.

## Supporting information

S1 Table PRISMA checklist. (DOC)

S2 Table Summarised extraction table. (DOCX)

S1 File Sample search string. (DOCX)

S2 File References of included studies. (DOCX)
