# Correction: Poverty and disability in low- and middle-income countries: A systematic review

**DOI:** 10.1371/journal.pone.0204881

**Published:** 2018-09-26

**Authors:** Lena Morgon Banks, Hannah Kuper, Sarah Polack

There are errors in Table 2. Some of the values in the section “Income group” are incorrect. Please see the corrected Table 2 here.

**Table 2:** Characteristics of included studies.

		Number^a^	%
***Region***	East Asia/Pacific	40	27
	Latin America/Caribbean	31	21
	South Asia	26	17
	Sub-Saharan Africa	22	15
	Middle East/North Africa	11	7
	Europe/Central Asia	4	3
	Multi-region	16	11
***Disability type***^***b***^	Visual impairment	12	8
	Hearing impairment	2	1
	Physical impairment	12	8
	Intellectual/cognitive impairment	23	15
	Mental disorders	73	49
	Mixed impairments/functional limitations	37	25
***Disability measure***	Impairment	114	76
	Activity/functional limitations	32	21
	Mixed	4	3
***Location***	Rural	17	11
	Urban	50	33
	Both	83	55
***Study design***	Cross-sectional	124	83
	Case-control	11	7
	Cohort	13	9
	Other	2	1
***Setting***	Community-based	133	89
	Hospital/clinic-based	6	4
	Schools	9	6
	Other	2	1
***Sample size***^***a***^	Smallest	85	
	First quartile (25^th^ percentile)	1,188	
	Median (50^th^ percentile)	3,591	
	Third quartile (75^th^ percentile)	10,667	
	Largest	2,600,000	
***Age group***^***c***^	Children	23	15
	Adults	41	27
	Older adults	48	33
	Mixed ages	38	25
***Income group***	Low	38	25
	Lower-middle	49	33
	Upper-middle	45	30
	Mix	18	12
***Poverty indicator***^***b***^	Income	82	55
	SES	36	24
	Assets	30	20
	PCE	10	7
	Other	2	1
***Risk of bias***	Low	81	54
	Medium	69	46

There are errors in Table 3. In the section “Income group,” the corresponding Null value for “Low” should be 36% and the corresponding Negative value for “Low” should be 4%. Please see the corrected Table 3 here.

**Table 3:** Association of poverty and poverty by study characteristics.

		Association of poverty with disability				
		*Positive*	*Null*	*Negative*	*Mixed*	*Total*
		%	%	%	%	N
**Overall**		81%	16%	2%	1%	150
**Disability/ impairment type**^a^	Sensory impairment	78%	17%	0%	6%	18
	Physical impairment	80%	15%	5%	0%	20
	Intellectual/cognitive impairment	69%	31%	0%	0%	26
	Mental disorders	87%	11%	3%	0%	75
	General disability/functional limitations	80%	14%	3%	3%	35
**Disability measure**^a^	Impairments	81%	16%	3%	1%	115
	Activity/functional limitations	79%	19%	0%	3%	33
	Mix	100%	0%	0%	0%	3
**Age group**^a^	Children	78%	15%	0%	7%	27
	Adults	86%	12%	2%	0%	42
	Older adults	69%	27%	4%	0%	49
	Mixed ages	92%	8%	0%	0%	36
**Poverty indicator**^a^	Income	83%	16%	0%	1%	82
	SES	81%	14%	3%	3%	36
	Assets	77%	20%	3%	0%	30
	PCE	60%	30%	10%	0%	10
**Region**^a^	Latin America/Caribbean	60%	30%	10%	0%	60
	East Asia/Pacific	80%	17%	2%	2%	60
	Sub-Saharan Africa	51%	44%	4%	0%	68
	South Asia	79%	19%	2%	0%	42
	Middle East/North Africa	87%	7%	7%	0%	15
	Europe/Central Asia	36%	64%	0%	0%	14
	Multi-region	100%	0%	0%	0%	8
**Income group**^a^	Low	59%	36%	4%	1%	95
	Lower-middle	67%	28%	5%	0%	100
	Upper-middle	72%	26%	1%	0%	69
**Gender**^a^	Female	87%	14%	0%	0%	30
	Male	86%	14%	0%	0%	22
**Setting**	Rural	82%	18%	0%	0%	17
	Urban	79%	19%	2%	0%	52
	Both	82%	12%	2%	2%	82
**Risk of bias**	Low	88%	10%	1%	1%	81
	Medium	74%	22%	3%	1%	69

^a^ Findings within studies have been disaggregated, where possible
