# Supplementary material for: Poverty and disability in low- and middle-income countries: A systematic review
Source: PLoS One. 2017 Dec 21;12(12):e0189996. doi: 10.1371/journal.pone.0189996 (PMC5739437; doi:10.1371/journal.pone.0189996)
Supplement: S2 Table — (DOCX) [file pone.0189996.s002.docx]

| **Citation** | | **Study design** | | **Study location (region, income)** | | **Rural/ urban** | | **Sample size** | | **Disability specifics and measure** | **Economic measure** | | Adjusted | | Overview of results | | **Summary of poverty and disability** | **Summary disability and work** | **Risk of bias (sources of bias)** |  |  |
| --- | --- | --- | --- | --- | --- | --- | --- | --- | --- | --- | --- | --- | --- | --- | --- | --- | --- | --- | --- | --- | --- |
| **SENSORY (HEARING, VISION)** | | | | | | | | | | | | | | | | | | |  |  |  |
| *ALL AGES* | | | | | | | | | | | | | | | | | | |  |  |  |
| Ataguba et al (2011)* | | CS (population-based) | | South Africa  (SSA, UM) | | Both | | – | | VI (self-reported) | SES | | Yes | | Prevalence of VI was disproportionately concentrated among lower SES quintiles (p<0.01) | | Positive | – | Low |  |  |
| Béria et al (2007) | | CS (population-based) | | Brazil  (LAC, LM) | | Urban | | 2,445 | | Disabling hearing impairment (CE, ≥41 dB (age ≥15 years), ≥31 dB (<15 years) in better ear) | Income | | Yes | | Prevalence of disabling hearing impairment was higher among individuals with incomes below US$200 compared to those above this threshold (OR =1.55; aOR=1.56 (95% CI: 1.06-2.27)) | | Positive | – | Low |  |  |
| Dandona et al (2001) | | CS (population-based) | | India  (SA, L) | | Both | | 10,293 | | Blindness (CE, VA <6/60) | SES | | Yes | | Increasing prevalence of blindness with worsening SES (p<0.0001); Upper vs extreme lower SES: aOR= 9.72 (95% CI: 2.30–41.0) | | Positive | – | Low |  |  |
| Dandona et al (2002) | | CS (population-based) | | India  (SA, L) | | Both | | 10,293 | | Moderate VI(CE, VA<6/18-6/60) | Income | | Yes | | Increasing prevalence of blindness with worsening SES (p=0.002): Upper vs extreme lower: 3.03 (95% CI: 1.78 - 5.17) | | Positive | – | Low |  |  |
| Freeman et al (2013) | | CS (population-based) | | Mix (70 countries) | | Both | | 260,958 | | VI (self-reported difficulty seeing) | Assets | | Yes | | In low income countries, prevalence of VI lower in highest asset group compared to lowest (aOR: 0.83, 95%CI: 0.72-0.95). Same for middle income countries (aOR: 0.0.69, 95% CI:0 0.61-0.78) | | Positive | – | Low |  |  |
| Habtamu et al (2015) | | CS (population-based) | | Ethiopia (SSA, L) | | Rural | | 200 | | VI (CE, VA<6/18) | Assets | | No | | Trichiasis cases were more likely to living in poverty if they had visual impairment, but not significant (OR: 1.71, 95% CI 0.98-2.97) | | Non-significant | – | Medium (no adjusting for confounding) |  |  |
| Ho et al (2001) | | Ecological (population-based) | | Mix (53 countries) | | Both | | – | | Blindness (CE, VA<3/60) | GNP | | Yes | | Prevalence of blindness is higher in developing countries with lower per capita income. | | Positive | – | Medium (potential for ecological fallacy) |  |  |
| Minh et al (2015) | | CS (population-based) | | Vietnam (EA, LM) | | Both | | 4,224 | | Vision/hearing (WGSS, at least “some difficulty” seeing) | Income | | Yes | | Households with members with visual impairment had “extra costs” of 12.7% (95% CI: 4.4-21%) of household income; for hearing 20.1% (9.5-30.6%) | | Positive | – | Low |  |  |
| *CHILDREN* | | | | | | | | | | | | | | | | | | |  |  |  |
| Natale et al (1992)* | | CS (population-based) | | India  (SA, L) | | Urban | | 640 | | Sensory (hearing and visual questions on Ten Questions Questionnaire) | Income | | No | | Children with sensory impairments were more likely to belong to the lowest social status group compared to the next to lowest (p=0.003) | | Positive | – | Medium (validity of economic measure) |  |  |
| Pham et al (2013) | | CS | | Vietnam (EA, L) | | Both | | 9,882 | | Vision (WGSS, at least “some difficulty” seeing) | Income | | Yes | | People with difficulty seeing were less likely to belong to the lowest income quintile compared to the highest (aOR=2.5, 95% CI: 1.39-4.66, p-trend: 0.001) | | Negative | – | Low |  |  |
| Taha et al (2010) | | CS (school-based) | | Egypt  (ME, LM) | | Both | | 555 | | Hearing impairment (CE, ≥20 dB) | SES | | No | | Children with hearing impairment more likely to be in moderate/low SES group compared to high (p<0.05) | | Positive | – | Medium (no adjustment, school-based, no response rate) |  |  |
| *ADULTS* | |  | |  | |  | |  | |  |  | |  | |  | |  |  |  |  |  |
| Emamian et al (2011) | | CS (population-based) | | Iran  (ME, UM) | | Urban | | 5,182 | | VI (CE, 0.3 LogMAR in better eye) | SES | | Yes | | Prevalence of VI increased with worsening SES (high: 3.6%, medium 7.5%, low 11.1%; p<0.001) | | Positive | – | Low |  |  |
| Emamian et al (2013) | | CS (population-based) | | Iran  (ME, UM) | | Urban | | 5,190 | | Near VI (CE, ≥1.6 M in better eye) | SES | | Yes | | Prevalence of VI increased with worsening SES (highest vs lowest: OR=3.05 (95% CI: 2.55-3.65), aOR=1.49 (1.20-1.86); highest vs. medium: OR=1.87 (1.55-2.26), aOR=1.2 (0.99-1.46) | | Positive | – | Low |  |  |
| Mathenge et al (2012) | | CS (population-based) | | Kenya  (SSA, L) | | Both | | 4,314 | | Blindness (CE, VA<3/60) | Assets | | Yes | | No significant difference in asset ownership between people with and without blindness (aOR: 0.5, 95% CI 0.2-1.1, poorest to least poor) | | Non-significant | – | Medium (only age adjusted, number of blind in each asset category is low - <15 for 3) |  |  |
| Zainal et al (1998) | | CS (population-based) | | Malaysia (EA, UM) | | Rural | | 282 | | VI (CE, VA<6/18) | Income | | No | | Mean level of income was not significantly differently between adults with and without VI | | No-significant | – | Medium (no adjusting, small sample, lack of information on analyses) |  |  |
| *OLDER ADULTS* | | | |  | |  | |  | |  |  | |  | |  | |  |  |  |  |  |
| Cockburn et al (2012) | | CS (population-based) | | South Africa  (SSA, UM) | | Urban | | 2,747 | | Vision impairment (CE, VA <6/18) | SES | | Yes | | Prevalence of VI increased with decreasing SES (aP<0.001); poorest to wealthiest SES tertile: OR= 4.5 (95% CI: 1.3-3.9); aOR=3.9 (95%CI: 2.2-6.7) | | Positive | – | Low |  |  |
| Kuper et al (2008) | | CC (population-based) | | Kenya  (SSA, L), Philippines (EA, LM), Bangladesh (SA, L) | | Both | | 1,131 | | VI due to cataract (CE, VA<6/24 in better eye) | 1. PCE 2. SES 3. Self-rated wealth | | Yes | | Increasing prevalence of VI with worsening PCE (test for trend of aORs: Kenya p=0.006, Bangladesh p=0.06, Philippines p=0.002); people with VI were more likely than people without VI to be in the lowest (poorest) quartile of PCE rather than highest (Kenya: aOR= 3.2, 95% CI: 1.2–8.8; Bangladesh: aOR=1.7 95% CI: 1.0–3.0; Philippines: aOR=2.4, 95% CI: 1.2–4.7);  Same pattern for SES index and self-rated wealth | | Positive | – | Low |  |  |
| Ploubidis et al (2013) | | CS (population-based) | | Kenya | | Both | | 1,402 | | VI (CE, VA<6/18 in best eye, available correction) | Assets | | Yes | | Older adults with VI owned significantly fewer assets than older adults without VI in rural areas; no significant difference in urban areas. | | Positive, mixed association | – | Low |  |  |
| Study design abbreviations: CC=case control, CS=cross-sectional; Means of assessment abbreviations: CE=clinical evaluation, dB=decibel, VA=visual acuity, VI=visual impairment, WGSS=Washington Group Short Set; Study location: EA=East Asia & Pacific, LAC=Latin America and the Caribbean, ME=Middle East & North Africa, SA=South Asia, SSA=Sub-Saharan Africa, L = low income country, LM=lower middle income country, UM=upper middle income country; Economic measure abbreviation: GNP=gross national product, PCE=per capita expenditure, SES=socioeconomic status; Overview of results abbreviations: CI=confidence interval, OR=odds ratio, aOR=adjusted odds ratio; *study is repeated in more than one category (results have been disaggregated by disability type) | | | | | | | | | | | | | | | | | | |  |  |  |
| ***Table 1:*** *Summary of studies examining sensory impairments and poverty* | | | | | | | | | | | | | | | | | | |  | |  |
| **Citation** | | **Study design** | | **Study location (region, income)** | | **Rural/ urban** | | **Sample size** | **Disability specifics and measure** | | **Economic measure** | | **Adjusted** | | **Association between disability and poverty** | **Summary of poverty and disability** | **Summary disability and work** | **Risk of bias (sources of bias)** | |  |  |
| *ALL AGES* | |  | |  | |  | |  |  | |  | |  | |  |  |  |  | |  |  |
| Ataguba et al (2011)* | | CS (population-based) | | South Africa (SSA, UM) | | Both | | – | Physical impairment (self-reported) | | SES | | Yes | | Prevalence of physical impairment was disproportionately concentrated among lower SES quintiles (p<0.01) | Positive | – | Low | |  |  |
| Lin et al (2013)^5^ | | CS (population-based) | | China (EA, LM) | | Both | | 2.6 million | Physical impairment caused by road traffic accidents (CE, ICF, ICD-10) | | Income | | Yes | | Adults: higher prevalence of disability from road traffic accidents among persons with lower family income (aOR= 1.61 (95% CI: 1.43–1.81). Children - no significant difference by income level | Positive | Positive | Low | |  |  |
| Minh et al (2015)* | | CS (population-based) | | Vietnam (EA, LM) | | Both | | 4,224 | Movement (WGSS, at least “some difficulty” seeing) | | Income | | Yes | | Households with members with movement difficulty had “extra costs” of 9% (0.5-17.5%) | Positive | – | Low | |  |  |
| Rischewski et al (2008) | | CC (population-based) | | Rwanda (SSA, L) | | Both | | 877 | Musculoskeletal impairment (CE, ICF definitions) | | 1. PCE  2. SES | | Yes | | No significant difference in PCE or SES among cases with and without physical impairment, except for with boys under 15 years (aOR: 2.2, 95% CI: 1.0-4.4) | Positive | Positive | Low | |  |  |
| Sozmen & Unal (2014)* | | CS (population-based) | | Turkey (ME, UM) | | Both | | 14,433 | Arthritis (self-report) | | Income | | Yes | | Arthritis prevalence increased with decreasing wealth (relative index of inequality: 1.36, 1.2-1.54) | Positive | Positive | Low | |  |  |
| Wang et al (2015c) | | CS (population-based) | | China (EA, UM) | | Both | | 21,435 | Arthritis (self-report of diagnosis) | | Income | | Yes | | Prevalence of arthritis increased with decreasing income level (lowest to highest group: aOR=0.70 (0.54-0.91) | Positive | – | Medium (validity of disability measure) | |  |  |
| *CHILDREN* | |  | |  | |  | |  |  | |  | |  | |  |  |  |  | |  |  |
| Ali et al (2013)* | | Cohort (population-based) | | Pakistan (SA, L) | | Urban | | 420 | Fine motor development | | Income | | Yes | | Children from households living below 3,500 rupees/month were more likely to experience delay (aOR: 2.2, 95% CI: 1.4-3.5) | Positive | – | Medium (low response rate, large loss to follow-up) | |  |  |
| Jiang et al (2013)* | | Cohort (population-based) | | Bangladesh (SA, L) | | Urban | | 398 | Motor development (Bayley Scales of Infant and Toddler Development) | | Income | | Yes | | Motor scores decreasing with decreasing family income (p=0.007) | Positive | – | Medium (large loss to follow-up, sampling unclear) | |  |  |
| Natale et al (1992)* | | CS (population-based) | | India  (SA, L) | | Urban | | 640 | Neuromotor (Ten Questions Questionnaire) | | Income | | No | | Children with neuromotor impairments were more likely to belong to the lowest social status group compared to the next to lowest (p=0.005) | Positive | – | Medium (validity of economic measure, no adjusting) | |  |  |
| Pham et al (2013)* | | CS (population-based) | | Vietnam (EA, L) | | Both | | 9,882 | Mobility (WGSS, at least “some difficulty”) | | Income | | No | | No difference in household income quintile people with and without mobility limitations (p-trend: 0.5) | Non-significant | – | Medium (lack of adjusting) | |  |  |
| *ADULTS* | |  | |  | |  | |  |  | |  | |  | |  |  |  |  | |  |  |
| Cordeiro de Andre et al (2015) | | CS (population-based) | | Brazil (LAC, UM) | | Both | | 18,745 | Mobility (7 questions on functional limitations) | | Income | | Yes | | No difference in prevalence of mobility limitations between income tertiles | Non-significant | Positive | Low | |  |  |
| Hosseinpoor et al (2012)* | | CS (population-based) | | 41 countries | | Both | | 170,298 | Arthritis (WHS questionnaire, symptom related questions) | | SES | | Yes | | Lower SES correlated with higher prevalence of arthritis (Men: significant in 2/4 models; Women: significant in 1/4 models) | Positive | – | Low | |  |  |
| Kilzieh (2010) | | CS (population-based) | | Syria (ME, LM) | | Urban | | 2,038 | Moderate/severe physical impairment (WHS Questionnaire, Health State Descriptions) | | SES | | Yes | | Higher prevalence of physical impairment in poorer SES group Moderate impairment: aOR 1.76 (95%CI: 1.09–2.84) Severe impairment: aOR 2.48 (95% CI: 1.32–4.67) | Positive | Non-significant | Low | |  |  |
| Vukovic et al (2008) | | CS (population-based) | | Serbia (Eur, UM) | | Both | | 14,552 | Arthritis (self-report) | | SES | | Yes | | People with arthritis were more likely to belong to the lowest wealth quintile compared to the highest: aOR (men) = 1.59 (95% CI: 1.25-2.02); aOR (women) = 1.41 (95% CI: 1.17-1.72) | Positive | – | Medium (validity of disability measure) | |  |  |
| *OLDER ADULTS* | | | |  | |  | |  |  | |  | |  | |  |  |  |  | |  |  |
| Blay et al (2012)* | | CS (population-based) | | Brazil (LAC, UM) | | Both | | 6,963 | Arthritis (self-reported, yes/no to sought treatment in last 6 months) | | Income | | Yes | | Prevalence of arthritis was higher in individuals below the poverty threshold compared to individuals at or above it, but this association was not significant after adjustment | Non-significant | Non-significant | Medium (validity of disability measure) | |  |  |
| Falkingham et al (2011) | | CS (population-based) | | Kenya, (SSA, L) | | Urban | | 2,037 | Mobility limitations (WHODAS) | | SES | | Yes | | Adults with mobility limitations were more likely to be from low wealth groups compared to high (p<0.01) | Positive | Non-significant | Low | |  |  |
| Fillenbaum et al (2010) | | CS (population-based) | | Brazil (LAC, UM) | | Urban | | 6,958 | Mobility (limitations in activities of daily living) | | Income | | Yes | | People with mobility limitations less likely to have a monthly income over US$200 (aOR: 0.7, 95% CI: 0.5-0.9) | Positive | – | Medium (validity of disability measure) | |  |  |
| Melzer et al (2004) | | CS (population-based) | | Brazil (LAC, UM) | | Both | | 28,943 | Mobility (Questionnaire - difficulties with daily physical activities) | | Income | | Yes | | Lower prevalence of disability in wealthier income groups. Highest vs lowest: aOR (M) = 0.43 (95% CI: 0.35-0.53), aOR (F): 0.72 (95%CI: 0.61-0.84) | Positive | – | Low | |  |  |
| Nakua et al (2015) | | CS (population-based) | | Ghana (SSA, L) | | Both | | 4,724 | Arthritis (self-report of diagnosis) | | Assets | | Yes | | People with arthritis were more likely to belong to the wealthiest quintile compared to the poorest (aOR: 1.95, 95% CI: 1.33-2.85) | Negative | – | Medium (validity of disability measure) | |  |  |
| Wu et al (2013)* | | CS (population-based) | | China  (EA, UM) | | Both | | 13,157 | Arthritis (self-report) | | SES | | Yes | | There was no difference in prevalence of arthritis between the lowest and highest quintile of SES | Non-significant | – | Medium (validity of disability measure) | |  |  |
| Study design abbreviations: CC=case control, CS=cross-sectional; Means of assessment abbreviations: CE=clinical evaluation, ICD-10: International Classification of Diseases, ICF: International Classification of Functioning, Disability and Health, WHS: World Health Survey WGSS=Washington Group Short Set; Study location: EA=East Asia & Pacific, Eur=Europe & Central Asia, LAC=Latin America and the Caribbean, ME=Middle East & North Africa, SA=South Asia, SSA=Sub-Saharan Africa, L = low income country, LM=lower middle income country, UM=upper middle income country; Economic measure abbreviation: PCE=per capita expenditure, SES=socioeconomic status; Overview of results abbreviations: OR=odds ratio, aOR=adjusted odds ratio, CI=confidence interval | | | | | | | | | | | | | | | | | |  | |  |  |
| ***Table 2:*** *Summary of studies examining physical impairments and poverty* | | | | | | | | | | | | | | | | | |  | |  |  |

| **Citation** | **Study design** | **Study location (region, income)** | **Rural/ urban** | **Sample size** | **Disability specifics and measure** | **Economic measure** | **Adjusted** | **Overview of results** | **Summary of poverty and disability** | **Summary disability and work** | **Risk of bias (sources of bias)** |
| --- | --- | --- | --- | --- | --- | --- | --- | --- | --- | --- | --- |
| *ALL AGES* | | | | | | | | | | |  |
| Ataguba et al (2011)* | CS (population-based) | South Africa (SSA, UM) | Both | – | Intellectual disability (self-reported) | SES | Yes | Prevalence of intellectual disability was disproportionately concentrated among lower SES quintiles (p<0.05) | Positive | – | Low |
| Minh et al (2015)* | CS (population-based) | Vietnam (EA, LM) | Both | 4,224 | Remembering (WGSS, at least “some difficulty”) | Income | Yes | Households with members with remembering difficulty had “extra costs” of 21.1% (11.6-30.6%) | Positive | – | Low |
| Pham et al (2013)* | CS (population-based) | Vietnam (EA, L) | Both | 9,882 | Remembering (WGSS, at least “some difficulty”) | Income | No | People with difficulty remembering were more likely to belong to the lowest income quintile compared to the highest (p-trend: 0.001) | Positive | – | Medium (no adjusting) |
| *CHILDREN* |  |  |  |  |  |  |  |  |  |  |  |
| Escueta et al (2014)* | CC (population-based) | Mix (5 countries) | Both | 1,780 | Cognitive development delay (KABC-II) | Assets | Yes | Cognitive score increased with increasing wealth (p<0.01) | Positive | – | Low |
| Halpern et al (2008) | Cohort (2 x 1 yr, hospital births) | Brazil (LAC, UM) | Urban | 5,271 | Developmental delay (Denver II Screening Test) | Income | Yes | Prevalence of suspected delay increased with decreasing income (p<0.005); highest vs lowest income groups PR (1994): 1.6 (95% CI: 1.2-2.1); PR (2004): 1.4 (95% CI: 1.1-1.8) | Positive | – | Medium (hospital-based) |
| Jiang et al (2013)* | Cohort (population-based) | Bangladesh (SA, L) | Urban | 398 | Cognitive and language development (Bayley Scales of Infant and Toddler Development) | Income | Yes | Cognitive and language scores decreasing with decreasing family income (cognitive: p=0.04, language: 0.03) | Positive | – | Medium (large loss to follow-up, sampling unclear) |
| Kumar et al (1997) | CS (population-based) | India  (SA, L) | Rural | 3,746 | Developmental delay (NP battery, below 25th percentile) | Income | Yes | Higher prevalence of slower psychosocial development in poorer income group: OR=2.30 (95%CI: 1.73-3.05); aOR=1.82 p=0.011 | Positive | – | Medium (validity of economic measure) |
| Natale et al (1992)* | CS (population-based) | India  (SA, L) | Urban | 640 | Cognitive impairment (Ten Questions Questionnaire) | Income | No | Children with cognitive impairments were more likely to belong to the lowest social status group compared to the next to lowest (p=0.05) | Positive | – | Medium (validity of economic measure) |
| Pheula et al (2011) | CC (public schools) | Brazil  (LAC, UM) | Urban | 200 | ADHD-I (K-SADS-E, CE) | SES | Yes | No significant association | No significant association | – | Medium (school-based) |
| Xie et al (2008) | CS (population-based) | China  (EA, LM) | Both | 60,124 | Intellectual disability (DDST, Gesell Developmental Inventory) | Income | No | Higher prevalence of ID in children from poorer income group OR=9.54 (95%CI: 4.82-18.91) | Positive | – | Medium (no response rate, no adjusting) |
| Zheng et al (2012) | CS (population-based) | China  (EA, LM) | Both | 106,754 | Intellectual disability (DDST, Gesell Development Inventory, Vinland Social Maturity Scale) | Income | Yes | Higher prevalence of ID among children in poorer income groups (Mild ID , lowest vs highest: aOR=2.01 (95% CI 1.55-2.82); Severe ID aOR=3.00 (95% CI 2.19-4.12) | Positive | – | Low |
| *OLDER ADULTS* | | | | | | | | | | |  |
| Arguvanli et al (2015) | CS (population-based) | Turkey (Eur, UM) | Urban | 900 | Cognitive impairment (MMSE) | Income | Yes | There was no significant difference in income between people with and without cognitive impairment. | Non-significant | – | Medium (sampling strategy unclear and may lead to bias, economic measure unclear) |
| Chen et al (2011) | Cohort (population-based, 7.5 yr) | China  (EA, LM) | Both | 1,307 | Dementia, incident (AGECAT) | Income | Yes | Incidence of dementia was lower in individuals who reported poor vs satisfactory income, but the difference was not significant | Non-significant | – | Medium (validity of economic measure) |
| Chen et al (2012) | CS (population-based) | China  (EA, LM) | Both | 2,917 | Dementia, prevalent (GMS/AGECAT) | Income | Yes | Prevalence of dementia was higher among individuals who reported their income as vs satisfactory income, although this association was only significant in Anhui (aOR = 2.18 (95% CI: 1.35-3.51), not the 4 provinces | Positive | – | Medium (validity of economic measure) |
| Dorsi et al (2011) | CS (population-based) | Brazil (LAC, UM) | Urban | 1,692 | Cognitive impairment (MMSE) | Income | Yes | Prevalence of cognitive impairment was higher among people from the lowest income quartile compared to the highest: aOR=1.29 (95% CI: 1.09-1.52) | Positive | – | Low |
| Falkingham et al (2011)* | CS (population-based) | Kenya (SSA, L) | Urban | 2,037 | Cognition, self-reported functioning (WHODAS) | SES | Yes | Higher wealth status not associated with reporting less problems with cognition (p=ns) | Non-significant | Non-significant | Low |
| Fei et al (2009) | CS (population-based) | China  (EA, LM) | Urban | 6,192 | Cognitive impairment, no dementia (Interview and NP screens) | Income | Yes | Higher prevalence of cognitive impairment among people with lower income: OR=1.48 (95%CI: 1.25-1.75); aOR=ns | Non-significant | – | Low |
| Herrera et al (2002) | CS (population-based) | Brazil (LAC, UM) | Urban | 1,656 | Dementia (MMSE, PFAQ, CE) | SES | Yes | No significant association between dementia and SES was found | Non-significant | – | Medium (analysis unclear) |
| Keskinoglu (2006) | CS (population-based) | Turkey (Eur, UM) | Urban | 201 | Dementia (MMSE) | Income | Yes | Higher prevalence of dementia in poorer income group: OR=3.25 (95%CI: 1.21-8.76); aOR=ns | Non-significant | Positive | Medium (small sample, that is mostly poor) |
| Li et al (2015b) | CS (population-based) | India  (SA, LM) | Both | 250,752 | Dementia-associated disability (screen + CE) | Income | Yes | People with dementia were not more likely to be above the national average income compared to below (OR=0.92, CI: 0.77-1.10) | Non-significant | – | Low |
| Lopes et al (2007) | CS (population-based) | Brazil  (LAC, LM) | Urban | 1,145 | Cognitive and functional impairment (MMSE, FOME, IQCODE, B-ADL) | SES | Yes | Higher prevalence of CFI in lower SES, not significant after adjusting OR=4.00 (95% CI: 1.81–8.87), aOR=ns | Non-significant | – | Medium (low response rate) |
| Peres et al (2015) | CS (population-based) | Brazil (LAC, UM) | Urban | 1,705 | Severe cognitive impairment (MMSE) | Income | No | People with cognitive impairments were more likely to belong to the poorest compared to the highest quartile of household income (OR=4.9, 95%CI:2.7-8.8) | Positive | – | Medium (no adjusting) |
| Saha et al (2010) | CS (population-based) | India  (SA, L) | Rural | 179 | Cognitive impairment (MMSE) | Income | Yes | Higher prevalence of cognitive impairment among people with lower income: OR=2.32 (95%CI: 1.18-2.32); aP-value: 0.05 | Positive | – | Medium (small sample, reliability of disability measure) |
| Scazufca et al (2008) | CS (population-based) | Brazil  (LAC, LM) | Urban | 2005 | Dementia (10/66 Dementia Research Group dementia diagnostic tool) | Income | Yes | Prevalence of dementia increased with decreasing income (p<0.001); Lowest to highest income group: aOR 3.38 (1.63-6.98) aP for trend <0.001 | Positive | – | Low |
| Sengupta et al (2014) | CS (population-based) | India  (SA, L) | Both | 3,038 | Cognitive impairment (modified Hindi Mental State Examination) | Income | Yes | People with cognitive impairment were more likely to have a monthly per capita household income of less than Rs 1000 | Positive | Positive | Low |
| Singh et al (1999) | CS (population-based) | India  (SA, L) | Urban | 595 | Cognitive deficits (Author-made questionnaire) | SES | No | Higher prevalence of cognitive deficits in poorer socio-economic classes (p<0.01) | Positive | – | Medium (no adjusting, response rate unclear) |
| Sosa et al (2012) | CS (population-based) | 8 countries | Both | 15,376 | Mild cognitive impairment (NP battery) | Assets | Yes | Lower prevalence of mild cognitive impairment associated with ownership of more assets compared to less assets: aOR (pooled) = 0.88 (0.82-0.95) | Positive | – | Low |
| Study design abbreviations: CC=case control, CS=cross-sectional; Means of assessment abbreviations: AGECAT= Automated Geriatric Examination for Computer Assisted Taxonomy, B-ADL: basic activities of daily living, CE=clinical evaluation, DDST=Denver Developmental Screening Test, GMS= Geriatric Mental State, IQCODE=Informant Questionnaire on Cognitive Decline in the Elderly, MMSE=mini-mental state evaluation, PFAQ=Pfeffer Functional Activities Questionnaire WGSS=Washington Group Short Set; Study location: EA=East Asia & Pacific, Eur=Europe & Central Asia, LAC=Latin America and the Caribbean, ME=Middle East & North Africa, SA=South Asia, SSA=Sub-Saharan Africa, L = low income country, LM=lower middle income country, UM=upper middle income country; Economic measure abbreviation: SES=socioeconomic status; Overview of results abbreviations: CI=confidence interval, OR=odds ratio, aOR=adjusted odds ratio, PR=prevalence ratio, aPR=adjusted prevalence ratio; *study is repeated in more than one category (results have been disaggregated by disability type) | | | | | | | | | | |  |

***Table 3:*** *Summary of studies examining intellectual disability/cognitive impairments and poverty*

|  |
| --- |

| **Citation** | | **Study design** | | **Study location (region, income)** | | **Rural/ urban** | | **Sample size** | | **Disability measure** | | | **Economic measures** | | **Adjusted** | | **Overview of results** | | **Summary of poverty and disability** | | **Summary disability and work** | | **Risk of bias (sources of bias)** | | |  |
| --- | --- | --- | --- | --- | --- | --- | --- | --- | --- | --- | --- | --- | --- | --- | --- | --- | --- | --- | --- | --- | --- | --- | --- | --- | --- | --- |
| *ALL AGES* | |  | |  | |  | |  | |  | | |  | |  | |  | |  | |  | |  | | |  |
| Arokiasamy et al (2015)* | | CS (population-based) | | 6 countries | | Both | | 42,236 | | Difficulties in ADL (SAGE measures, based on WHODAS 2.0) | | | SES | | No | | SES negatively associated with having at least one limitation in ADL | | Positive | | – | | Medium (no adjusting, response rate low, analysis unclear) | | |  |
| Danquah et al (2014) | | CC (population-based) | | Haiti  (LAC, L) | | Urban | | 254 | | Functional limitations (WGSS, “some difficulty” in 2+ activities or “a lot”/”cannot do” for 1+) | | | Assets | | Yes | | People with functional limitations were not more likely to belong to the lowest vs highest SES quartile (aOR: 1.3, 95% CI: 0.7-2.3) | | Non-significant | | Positive | | Low | | |  |
| Ergin & Kunst (2015) | | CS (population-based) | | Turkey  (Eur, LM) | | Both | | 10,791 | | Functional limitations (WHS questions) | | | Assets | | Yes | | People with functional impairments were more likely to belong to the poorest wealth group compared to richest (various models, all p<0.05) | | Positive | | – | | Low (disability measure picks up more severe forms) | | |  |
| Filmer (2008) | | CS (population-based) | | 13 countries | | Both | | 891,466 | | All disability (National household surveys, disability definition varies) | | | SES | | Yes | | Children: positive and significant in 2/14 surveys (India, Indonesia); Adults: positive, significant in 8/12 surveys | | Positive | | – | | Medium (no response rates, disability measure varies, not always robust) | | |  |
| Hoogeveen (2005) | | CS (population-based) | | Uganda  (SSA, L) | | Both | | 447,498 | | General disability in head of household (Population and Housing Census 1991, disability: impairment preventing labour in past week) | | | PCE | | Yes | | Lower mean per capita expenditure among households with a disabled household head (significant difference in 3/4 regions); households with disabled head more likely to be below the poverty line (significant in 4/4 regions) | | Positive | | – | | Low | | |  |
| Minh et al (2015) | | CS (population-based) | | Vietnam (EA, LM) | | Both | | 4,224 | | Self-care, communication (WGSS, at least “some difficulty”) | | | Income | | Yes | | Households with members with remembering difficulty had “extra costs” of 10.5 (-5 to 26%) for self-care and 32.9% (17.6-48.2%) for communication | | Positive | | – | | Low | | |  |
| Mont & Nguyen (2011) | | CS (population-based | | Vietnam | | Both | | 36,645 | | Functioning (WGSS, “some difficulty” in 2+ activities, “a lot of difficulty” or “cannot do” in 1+ activities) | | | PCE | | Yes | | Households with a person with a disability are over-represented in the lower consumption quartiles (various models, most p<0.01) | | Positive | | Positive | | Low | | |  |
| Palmer et al (2012) | | CS (population-based) | | Vietnam  (EA, L) | | Both | | 60,737 | | Functional difficulties and ADL (questionnaire, ICF based) | | | Assets | | No | | People with disabilities were poorer than people without disabilities PR= 1.76 (severe: PR = 1.83); p<0.001 | | Positive | | – | | Medium (no adjusting) | | |  |
| Palmer et al (2014) | | CS (population-based) | | Vietnam  (EA, L) | | Both | | 390,070 | | Functional limitations (WGSS, “a lot” or “cannot do” to 1+ activity) | | | Income | | Yes | | People with disabilities were more likely to experience health care induced poverty compared to other target insurance groups (p<0.001) | | Positive | | – | | Medium (no adjusting) | | |  |
| Subbaraman et al (2014)* | | CS (population-based) | | India  (SA, LM) | | Urban | | 521 | | Functional limitations (WHODAS 2.0) | | | Income | | Yes | | People in the richest household income category were less likely to have functional limitations compared to those in the poorest (aOR: 0.31 (0.10-0.97) | | Positive | | – | | Low | | |  |
| Trani et al (2015b) | | CC (population-based) | | Morocco, Tunisia (ME, LM) | | Both | | 2,509 | | Activity limitations (Disability Screening Questionnaire) | | | SES  Assets | | Unclear | | People with disabilities were multidimensionally poorer in both countries compared to people without disabilities (p<0.001), also poorer when just considering assets | | Positive | | Positive | | Medium (methods unclear, including if adjusted for confounding) | | |  |
| *CHILDREN* | |  | |  | |  | |  | |  | | |  | |  | |  | |  | |  | |  | | |  |
| Dang et al (2016) | | CS (population-based) | | Vietnam  (EA, LM) | | Both | | 1,314 | | Functional impairment (Brief Impairment Scale) | | | Income | | Yes | | Increased prevalence of functional impairment in children with decreasing household income (p<0.01) | | Positive | | – | | Medium (no response rate; controlled for area, otherwise unclear) | | |  |
| Kawakatsu et al (2012) | | CS (population-based) | | Kenya  (SSA, L) | | Rural | | 339 | | Hearing, physical, visual, cognitive impairment and epilepsy (TQQ, CE, NP battery) | | | Income | | Yes | | Children with disabilities more likely to be in poorest income group compared to those without (OR=ns; aOR=2.79 (95%CI=1.28-6.08) | | Positive | | – | | Medium (validity of economic measure, small sample, sampling strategy may lead to bias) | | |  |
| Kumar et al (2013)* | | CS (population-based) | | India | | Both | |  | | Neurological disorders: epilepsy, global developmental delay, and motor, vision, and hearing) | | | Income  assets | | Yes | | Both asset ownership and income were lower among families with child with a disability, but this difference was only significant for asset ownership (p<0.001) | | Positive | | – | | Low | | |  |
| Kuper et al (2014) | | CS (program participants) | | 30 countries | | Both | | 898,834 | | Multiple types of impairments (parent-reported) | | | SES | | Yes | | 9/30 countries showed a positive association, 15/30 no association and 6/30 a negative association | | Mixed | | – | | Medium: limited adjustment, sampling may lead to bias | | |  |
| Kuper et al (2015) | | CC (by key informants) | | Kenya (SSA, L) | | Rural | | 807 | | Moderate/severe impairments (UNICEF-Washington Group questionnaire, confirmed CE) | | | Assets | | Yes | | There was no significant difference in asset score between households with and without a child with a disability (aOR: 0.8, 95% CI: 0.5-1.2) | | Non-significant | |  | | Low (not population-based, but sampling strategy validated) | | |  |
| Loyalka et al (2014) | | CS (population-based) | | China  (EA, LM) | | Both | | 2.5 million | | Mixed impairment types (screened and CE confirmation) | | | Income | | No | | Income of households with a person with a disability on average 2,150 yuan (one person) to 3427 yuan (2+ people with disabilities) less and this was significant | | Positive | | – | | Medium: no adjustment | | |  |
| Marella et al (2015) | | CS (population-based) | | Bangladesh  (SA, L) | | Both | | 1,855 | | Mixed impairment types (Rapid Assessment of Disability) | | | Assets | | Yes | | Compared to people in the highest wealth quintile, households in the bottom quintile were more likely to have a member with a disability (aOR=1.9, 95%CI: 1.09-3.3) | | Positive | | Positive | | Low | | |  |
| Natale et al (1992)* | | CS (population-based) | | India | | Urban | | 640 | | Serious disability (TQQ) | | | Income | | Yes | | Higher proportion of families with disabilities living in area with lowest family income compared to next lowest: aOR=2.39 (95% CI: 1.85-3.09) | | Positive | | – | | Medium: validity of economic measure | | |  |
| Ou et al (2015)* | | CC (school-based) | | China  (EA, UM) | | Both | | 1,301 | | Mixed impairments (self-report of clinical diagnosis) | | | Income | | Yes | | There was a significant difference between households with and without a child with disabilities (p<0.001) | | Positive | | – | | Medium (likely selection bias, controls not well matched on gender) | | |  |
| Pham et al (2013)* | | CS (population-based) | | Vietnam (EA, L) | | Both | | 9,882 | | Self-care, communication (WGSS, at least “some difficulty”) | | | Income | | No | | People with communication difficulty more likely to belong to lower household income groups (p-trend: 0.001); no difference for people with self-care difficulty | | Positive | | – | | Medium (no adjusting) | | |  |
| Trani et al (2013) | | CS (population-based) | | Afghanistan (SA, L) | | Both | | 1,184 | | Mixed impairment types (questionnaire based on ICF) | | | SES  Assets | | Unclear | | Children with disabilities more likely to belong to multidimensionally poorer households, as well as households with fewer assets | | Positive | |  | | Low | | |  |
| *ADULTS* | |  | |  | |  | |  | |  | | |  | |  | |  | |  | |  | |  | | |  |
| Hosseinpoor et al (2013) | | CS (population-based) | | 49 countries | | Both | | 218,737 | | Functioning (World Health Survey) | | | SES | | Yes | | Disability prevalence highest in poorest compared to richest wealth quintiles. Unadjusted: all positive but significant for 16/18 (LICs), 14/15 (lower MICs), 9/9 (upper MICs) Adjusted: all positive but significant for 9/18 (LICs), 7/15 (lower MICs), 7/9 (upper MICs) | | Positive | | – | | Low | | |  |
| Mitra et al (2013) | | CS (population-based) | | 15 countries | | Both | | 91,824 | | General disability - functional limitations (World Health Survey) | | | 1. PCE  2. Assets | | Yes | | 1. Higher proportion of households with disabilities under the extreme poverty line compared to households without disabilities, significant in 3/15 countries 2. Households with disability are more lively to be asset deprived in 12/15 countries but only statistically significant in 4/15 in countries | | Positive | | Positive | | Low | | |  |
| Trani et al (2012) | | CC (nested, population-based) | | Afghanistan (SA, L) ,Zambia (SSA, L) | | Both | | 5,032 | | General disability (questionnaire, ICF based and WGSS) | | | Assets | | Yes | | Asset ownership not significantly different between people with and without disabilities | | Non-significant | | Positive | | Low | | |  |
| *OLDER ADULTS* | | | |  | |  | |  | |  | | |  | |  | |  | |  | |  | |  | | |  |
| Basu & King (2013) | | CS (population-based) | | India  (SA, LM) | | Both | | 7,150 | | Functional limitations (WHODASi 2.0) | | | Income | | Yes | | Wealth inversely associated with functional limitations (p<0.001) | | Positive | |  | | Low | | |  |
| Beydoun et al (2005) | | Cohort (population-based, 3 years) | | China  (EA, LM) | | Both | | 976 | | Functional status decline: ADL (IADL, modified Katz questionnaire) | | | Income | | Yes | | Incidence of functional status decline increased with decreasing household income (adjusted for age/gender), but not significant after controlling for rural-urban residence and living arrangements). | | Positive | | – | | Low (slightly high loss to follow-up, 26%) | | |  |
| Falkingham et al (2011)* | | CS (population-based) | | Kenya  (SSA, L) | | Urban | | 2,037 | | Self-reported functioning – self-care, interpersonal and life activities (WHODAS) | | | SES | | Yes | | Higher wealth status associated with reporting less disability (p<0.001) | | Positive | | Positive | | Low | | |  |
| Fillenbaum et al (2010) | | CS (population-based) | | Brazil  (LAC, UM) | | Urban | | 6,958 | | Limitations in ADL -help needed with daily activities (self-reported) | | | Income | | Yes | | Individuals with incomes below US$200 reported more limitations in ADL (OR significant in 5/5 categories; aOR significant for 3/5) | | Positive | | – | | Medium (validity of disability measure) | | |  |
| Guerra et al (2008) | | CS (population-based) | | Brazil  (LAC, UM) | | Urban | | 2,143 | | Disability in ADL (questionnaire, self-reported) | | | Income | | Yes | | Perceived insufficient current income (aOR=1.91, 95% CI: 1.49-2.45) and poor childhood economic situation (aOR=1.29, 95% CI: 1.02-1.64) were both associated with higher prevalence of disability in ADL. | | Positive | | – | | Medium (validity of economic and disability measures) | | |  |
| Gureje et al (2006)^91^ | | CS (population-based) | | Nigeria  (SSA, L) | | Both | | 2,152 | | Disability in ADL and IADL (Katz index, Nagi scale) | | | Assets | | Yes | | No significant association between asset ownership and disability in ADL or IADL. | | Non-significant | | – | | Medium (limited adjustment for confounders) | | |  |
| Liu et al (2009) | | CS (population-based) | | China  (EA, LM) | | Both | | 354,857 | | Functional disability, mobility focused (CE, using ICF criteria) | | | Income | | Yes | | Higher prevalence of disability in poorest compared to richest income group (OR=2.166, 95%CI: 2.075-2.262) | | Positive | | Positive | | Low | | |  |
| Razzaque et al (2010) | | CS (population-based) | | Bangladesh  (SA, L) | | Rural | | 4,000 | | Functional ability (WHODASi) | | | SES | | Yes | | Poorer functional ability scores in lower SES groups | | Positive | | – | | Low | | |  |
| Wandera et al (2014) | | CS (population-base) | | Uganda (SSA, L) | | Both | | 2,382 | | Activity limitations (WGSS, “some difficulty” in 2+ activities, “a lot of difficulty” or “cannot do” in 1+ activities) | | | PCE | | Yes | | Household per capita consumption was not significantly associated with having a member with a disability. | | Non-significant | | – | | Low | | |  |
| Williams et al (2015) | | CS (population-based) | | 6 countries | | Both | | 29,807 | | Functional limitations (WHODAS 2.0) | | | Assets | | Yes | | Prevalence of disability increased with decreasing wealth status (p<0.01) | | Positive | |  | | Low | | |  |
| Xavier Gómez-Olivé (2010) | | CS (population-based) | | South Africa (SSA, UM) | | Urban | | 4,085 | | Functional limitations (WHODAS) | | | Assets | | Yes | | Higher prevalence of disability in poorest compared to wealthiest group OR = 1.24 (95% CI: 1.03 - 1.50) | | Positive | | Positive | | Medium (low response rate, particularly of men; exclusion of people with hearing impairment) | | |  |
| Study design abbreviations: CC=case control, CS=cross-sectional; Means of assessment abbreviations: ADL= activities of daily living, CE=clinical evaluation, IADL: instrumental activities of daily living, ICF: International Classification of Functioning, Disability and Health, NP:=neuropsychological, TQQ=Ten Questions Questionnaire, WHODAS=WHO Disability Assessment Schedule, WHODASi: WHODAS inverted; Economic measure abbreviation: PCE=per capita expenditures, SES=socioeconomic status; Overview of results abbreviations: CI=confidence interval, OR=odds ratio, aOR=adjusted odds ratio | | | | | | | | | | | | | | | | | | | | | | |  |  |  |  |
| ***Table 5:*** *Summary of studies examining reported functional limitations, general disability* | | | | | | | | | | | | | | | | | | | | | | |  |  | | |
| **Citation** | | **Study design** | | **Study location** | | **Rural/ urban** | | **Age group** | | **Disability measure** | **Economic measure** | | **Adjusted** | | **Overview of results** | | **Summary of poverty and disability** | | **Summary disability and work** | | **Risk of bias (source of bias)** | | |  |  |  |
| **DEPRESSION & ANXIETY** | | | | | | | | | | | | | | | | | | | | |  | | |  |  |  |
| *ALL AGES* | | | | | | | | | | | | | | | | | | | | |  | | |  |  |  |
| Arokiasamy et al (2015)* | | CS (population-based) | | 6 countries | | Both | | 42,236 | | Difficulties in ADL (SAGE measures, based on WHODAS 2.0) | SES | | No | | SES negatively associated with having depression | | Positive | | – | | Medium (no adjusting, low response rate, analysis unclear) | | |  |  |  |
| Liu et al (2015a) | | CS (population-based) | | China  (EA, UM) | | Both | | 16,032 | | Major depressive disorder (SCID administered by clinician) | Income | | Yes | | People were more likely to have major depressive disorder if they were from lower income groups (p=0.002) | | Positive | | Positive | | Low | | |  |  |  |
| Zhou et al (2015) | | CS (population-based) | | China  (EA, UM) | | Rural | | 11,473 | | Depression (PHQ-9) | Income | | Yes | | Lower income associated with depressive symptoms (p<0.001); aOR high to low income: 0.418, 95% CI: 0.32-0.54 | | Positive | | – | | Low | | |  |  |  |
| *CHILDREN* | | | | | | | | | | |  | |  | |  | |  | |  | |  | | |  |  |  |
| Wang et al (2015b) | | CS (school-based) | | China  (EA, UM) | | Rural | | 4,857 | | Depression (Children’s Depression Inventory) | Income | | Yes | | Among children whose parents had migrated to urban centres, high household income was protective against depressive symptoms (aOR=1.56, 95% CI: 1.25-1.93 for lowest to highest income group. | | Positive | | – | | Medium (school-based, sample not generalizable) | | |  |  |  |
| *ADULTS* | |  | |  | |  | |  | |  |  | |  | |  | |  | |  | |  | | |  |  |  |
| Abas et al (1997) | | CS (population-based) | | Zimbabwe (SSA, L) | | Urban | | 172 | | Depression and anxiety (Shona Screen for Mental Disorders, Present State Examination) | Income | | Yes | | Prevalence of depression/anxiety was higher in women with below average income compared to women with above average income (OR=2.22, 95% CI: 1.06-4.67); aOR=ns) | | No significant association | | – | | Medium (small sample size, validity of economic measure) | | |  |  |  |
| Ball et al (2010) | | CS (population-based) | | Sri Lanka (SA, LM) | | Both | | 5,968 | | Depression (CIDI) | SES | | Yes | | Lifetime prevalence of depression was higher in individuals from the poorest 2 quintiles of standard of living compared to those from the riches 3 quintiles (OR=1.33 (95%CI: 1.12–1.57), aOR=1.25 (95%CI 1.05–1.49)). | | Positive | | – | | Low | | |  |  |  |
| Chen et al (2013) | | CS (university students) | | China  (EA, LM) | | Both | | 5,242 | | Depression (Beck Depression Inventory) | Income | | Yes | | Prevalence of depression higher among students from poor compared to good family economic situation (OR =1.80 95% CI: 1.51-2.15; aOR = 1.34 95% CI: 1.13-1.58) | | Positive | | – | | Medium: school-based, potential selection bias | | |  |  |  |
| Hosseinpoor et al (2012)* | | CS (population-based) | | 41 countries | | Both | | 170,298 | | Depression (World Health Survey questionnaire, ICD-10) | SES | | Yes | | Lower SES correlated with higher prevalence of depression (Men: significant in 4/4 models; Women: significant in 3/4 models) | | Positive | | – | | Low | | |  |  |  |
| Ibrahim et al (2012) | | CS (university students) | | Egypt  (ME, L) | | Both | | 1,366 | | Depression (Zagazig Depression scale - based on Hamilton Rating Scale) | Income | | Yes | | Lower prevalence of depression associated with higher income | | Positive | | – | | Medium (specificity of disability measure, school-based) | | |  |  |  |
| Ma et al (2009) | | CS (population-based) | | China  (EA, LM) | | Both | | 5,926 | | General anxiety disorder, lifetime prevalence (CIDI, ICD-10) | Income | | Yes | | No association between income and general anxiety disorder | | Non-significant | | Non-significant | | Low | | |  |  |  |
| Sozmen & Unal (2014)* | | CS (population-based) | | Turkey (ME, UM) | | Both | | 14,433 | | Depression (self-report) | Income | | Yes | | Depression prevalence increased with decreasing wealth (relative index of inequality: 1.99 (1.44-2.75) | | Positive | | – | | Low | | |  |  |  |
| Topuzoglu et al (2015) | | CS (population-based) | | Turkey  (ME, UM) | | Both | | 4,011 | | Clinical major depressive disorder (CIDI 2.1, sect E) | Income | | Yes | | Clinical depression increased with decreasing monthly income, but not significantly (high to low income: aOR=1.4 (0.9-2.2) | | Non-significant | | Positive | | Low | | |  |  |  |
| Vukovic et al (2008) | | CS (population-based) | | Serbia (Eur, UM) | | Both | | 14,552 | | Anxiety and depression (self-report) | SES | | Yes | | People with depression were more likely to belong to the lowest wealth quintile compared to the highest: aOR (men) = 2.98 (95% CI: 1.68-5.26); aOR (women) = 1.56 (95% CI: 1.06) | | Positive | | – | | Medium (validity of disability measure) | | |  |  |  |
| Weobong et al (2014) | | Cohort (population-based) | | Ghana  (SSA, L) | | Both | | 21,135 | | Antenatal depression (PHQ-9) | Assets | | Yes | | Women who were poorer were more likely to have antenatal depression (p=0.015, aOR lowest to highest: 1.30, 95% CI: 1.09-1.55) | | Positive | | – | | Low | | |  |  |  |
| Wu et al (2013)* | | CS (population-based) | | China  (EA, UM) | | Both | | 13,157 | | Depression (self-report) | SES | | Yes | | There was no difference in prevalence of depression between the lowest and highest quintile of SES | | Non-significant | | – | | Medium (validity of disability measure) | | |  |  |  |
| Wu et al (2014) | | CS (population-based) | | China  (EA, LM) | | Urban | | 2,080 | | Anxiety, depression, PTSD (SRQ-20) | Income | | Yes | | People with lower incomes more likely to experience psychological symptoms (p<0.01) | | Positive | | – | | Low | | |  |  |  |
| *OLDER ADULTS* | | | |  | |  | |  | |  |  | |  | |  | |  | |  | |  | | |  |  |  |
| Blay et al (2007) | | CS (population-based) | | Brazil  (LAC, UM) | | Both | | 6,961 | | Depression (Short Psychiatric Evaluation Schedule) | Income | | Yes | | Prevalence of depression was significantly higher in individuals with incomes below the poverty threshold compared to individuals at or above it OR=2.19 (95%CI: 1.97-2.43); aOR=1.53 (95% CI: 1.35-1.75). | | Positive | | Positive | | Medium (sampling methods unclear) | | |  |  |  |
| Chen et al (2005) | | CS (population-based) | | China  (EA, LM) | | Rural | | 1,600 | | Depression (GMS-AGECAT) | Income | | Yes | | Prevalence of depression was higher in older adults from the lowest income group compared to highest (OR=8.14 (95% CI: 4.13-16.06); aOR=2.49 (95% CI: 1.17-5.28). | | Positive | | – | | Medium (validity of economic measure) | | |  |  |  |
| Dasgupta et al (2013) | | CS (population-based) | | India  (SA, LM) | | Rural | | 85 | | Depression (Yesaverage’s Geriatric Depression Scale, short form) | Income | | Yes | | Depression was more prevalent in those living below Rs 1000 compared to above (aOR=7.6, 95%CI: 1.9-31.8) | | Positive | | – | | Medium (small sample size, response rate unclear) | | |  |  |  |
| Guerra et al (2009) | | CS (population-based) | | Peru, Mexico, Venezuela | | Both | | 5886 | | Depression (DSM-IV and ICD-10 criteria, GMS-AGECAT, EURO-D, ICD-10 depressive episode) | Assets | | Yes | | No significant association with number of household assets for any country, before or after adjustment. | | Non-significant | | – | | Medium (lack of information of study participants, analysis) | | |  |  |  |
| Guo et al (2014) | | CS (population-based) | | China  (EA, LM) | | Both | | 629 | | Depression (CES-D) | Income | | No | | People whose personal income was in the highest group were less likely to experience depression than those in the poorest group (aOR=0.4, 95%CI: 0.17-95) | | Positive | | – | | Medium (no adjustment, sensitivity of economic measure) | | |  |  |  |
| Gureje et al (2007) | | CS (population-based) | | Nigeria  (SSA, L) | | Both | | 1,897 | | Lifetime major depressive disorder (CIDI, DSM-IV) | Assets | | No | | Lower prevalence of depression in poorer SES groups. Highest vs lowest OR for = 0.5 (95%CI: 0.3-0.8)) | | Negative | | – | | Medium (limited adjustment) | | |  |  |  |
| Hanandita et al (2014) | | CS (population-based) | | Indonesia (EA, LM) | | Both | | 577,548 | | Depression (SRQ-20) | PCE | | Yes | | Likelihood of having depression increased with decreasing PCE (p<0.05) | | Positive | | Positive | | Low | | |  |  |  |
| Kulkarni & Shinde (2015) | | CS (population-based) | | India  (SA, LM) | | Both | | 7,150 | | Moderate/severe depression (ICD-10) | SES | | Yes | | People from the poorest SES group were more likely to experience depression compared to the richest (aOR=2.6, 95%CI: 1.7-3.9) | | Positive | | – | | Low | | |  |  |  |
| Fernandez-Nino et al (2015) | | CS (population-based) | | Mexico (LAC, UM) | | Both | | 8,874 | | Clinically significant depressive symptoms (CES-D) | Assets | | Yes | | People from the lowest tertile of asset ownership were more likely to experience depressive symptoms compared to those in the highest (p<0.01) | | Positive | | Non-significant | | Low | | |  |  |  |
| Lei et al (2014) | | CS (population-based) | | China  (EA, UM) | | Both | | 14,923 | | Depression (CES-D) | PCE | | Yes | | Increase in PCE associated with a decline in CES-D score (less depression) (p<0.05) | | Positive | | – | | Low | | |  |  |  |
| Li et al (2011) | | CS (population-based) | | China  (EA, LM) | | Both | | 1,921 | | Depression (GDS-15, score >7) | Self-rated wealth | | Yes | | People with depression more likely to be in the poorest economic group (OR= 17.69 (95%CI: 9.28–33.75); aOR=8.319 (p<0.001)) | | Positive | | Non-significant | | Medium (economic measure unclear) | | |  |  |  |
| Malhotra et al (2010) | | CS (population-based) | | Sri Lanka  (SA, LM) | | Both | | 999 | | Depression, clinically significant (GDS-15, score: ≥6) | Income | | Yes | | Higher prevalence of depression in lower income group Unadjusted = p<0.05, Adjusted (model 1)=p<0.05; (model 2) = 0.89 (95% CI: 0.76–1.04) | | Positive | | – | | Medium (economic measure, analysis unclear, limited adjusting) | | |  |  |  |
| Minicuci et al (2014) | | CS (population-based) | | Ghana  (SSA, L) | | Both | | 4,724 | | Depression (self-report of diagnosis) | SES | | No | | People with depression were more likely to below to the poorest compared to highest SES group (p<0.05) | | Positive) | | – | | Medium (no adjusting, validity of disability measure) | | |  |  |  |
| Rajkumar et al (2009) | | CS (population-based) | | India  (SA, LM) | | Rural | | 1,000 | | Depression (BMS,  WHODAS, CERAD, HAS-DSS, Neuropsychiatric Inventory) | Income | | Yes | | Higher prevalence of depression among people with lower family income OR=2.47 (95% CI: 1.65–3.68), aOR=1.78 (95% CI: 1.08-2.91) | | Positive | | – | | Low | | |  |  |  |
| Sengupta & Benjamin (2015) | | CS (population-based) | | India  (SA, LM) | | Both | | 3,038 | | Depression (GDS-15) | Income | | Yes | | People with incomes below Rs 1000 were more likely to experience depression compared to people at or above this threshold (aOR=2.17, 95%CI: 1.56-7.5) | | Positive | | Non-significant | | Low | | |  |  |  |
| **COMMON MENTAL DISORDERS** | | | | | | | | | | | | | | | | | | | | |  | | |  |  |  |
| *ALL AGES* | |  | |  | |  | |  | |  |  | |  | |  | |  | |  | |  | | |  |  |  |
| Jenkins et al (2015) | | CS (population-based) | | Kenya  (SSA, L) | | Rural | | 1,157 | | Common mental disorders (CIS-R) | Assets | | Yes | | People from the lowest wealth group were more likely to have a common mental disorder than if they were from the highest wealth group (aOR=2.5, 95% CI: 1.4-4.8) | | Positive | | Negative | | Low | | |  |  |  |
| Subbaraman et al (2014) | | CS (population-based) | | India  (SA, LM) | | Urban | | 521 | | Common mental disorders (GHQ-12) | Income | | Yes | | There was no difference in prevalence of common mental disorders between income groups. | | No significant assocation | | – | | Low | | |  |  |  |
| *ADULTS* | |  | |  | |  | |  | |  |  | |  | |  | |  | |  | |  | | |  |  |  |
| Anselmi et al (2008) | | Cohort (hospital-based) | | Brazil | | Urban | |  | | Common mental disorders (SRQ-20, minimum 8 symptoms) | Income | | Yes | | Prevalence of CMD higher for those whose family income at birth was in the lowest group compared to the highest group; prevalence of CMD was higher amongst individuals who were in the lowest tertile of family income throughout their life course compared to individuals who were consistently in the first and second tertiles. | | Positive | | – | | Low (hospital-based birth cohort) | | |  |  |  |
| Coelho et al (2009) | | CS (population-based) | | Brazil  (LAC, LM) | | Urban | | 1,327 | | Common mental disorders (SRQ-20, min 6 symptoms for women, 8 for men) | SES | | Yes | | Higher prevalence of CMD among poorer SES groups (p for trend <0.001). OR for poorest compared to wealthiest: OR=3.79 (95%CI: 2.34-6.14); aOR=3.33 (2.01-5.52) | | Positive | | Positive | | Low | | |  |  |  |
| Graham et al (2014) | | CS (population-based) | | Vietnam  Philippines Indonesia (EA, LM) | | Both | | 3,026 | | Common mental disorders (SRQ-20) | Assets | | Yes | | People in the highest asset group were less likely to have a common mental disorder than those who were in the poorest (p<0.05 in all countries) | | Positive | | – | | Low | | |  |  |  |
| Lima et al (1996) | | CS (population-based) | | Brazil  (LAC, UM) | | Urban | | 1,277 | | Common mental disorders (SRQ-20) | Income | | Yes | | Prevalence of CMD was higher in individuals from the poorest compared to richest tertile of family income (aOR= 2.25 (95% CI: 2.15–2.35)) | | Positive | | – | | Low | | |  |  |  |
| Ludermir et al (2001) | | CS (population-based) | | Brazil  (LAC, UM) | | Rural | | 621 | | Common mental disorders (SRQ-20) | Income | | Yes | | Higher prevalence of CMD among poorer income group OR=3.88 (95%CI: 2.1-7.1); aOR=2.4 (95% CI: 1.0-5.6) | | Positive | | – | | Low | | |  |  |  |
| Nguyen et al (2015) | | CS (population-based) | | Vietnam  (EA, L) | | Rural | | 211 | | Postpartum common mental disorders (SCID-I) | SES | | Yes | | Women in the lowest 25% of household had higher risk of CMD 1 year after birth (aOR=4.3, 95%CI: 1.2-15.3) compared to women in the highest 75% of households | | Positive | | – | | Medium (sample size small) | | |  |  |  |
| Patel et al (2006) | | Cohort (population-based, 1 year) | | India  (SA, L) | | Both | | 2,166 | | Common mental disorders, incident (Revised Clinical Interview Schedule, Scale for Somatic Symptoms) | Income | | Yes | | Increasing incidence of CMD with decreasing income (aP-for-trend p=0.04) | | Positive | | – | | Low | | |  |  |  |
| Quadros et al (2015) | | Cohort (hospital-based) | | Brazil  (LAC, UM) | | Urban | | 3,642 | | Common mental disorders (SRQ-20) | Income | | No | | Prevalence of CMD highest in those who were poor over the 3 time periods and lowest in those who were non-poor over all 3 (p<0.001) | | Positive | | – | | Medium (hospital-based, 32% loss to follow-up, no adjusting) | | |  |  |  |
| Rocha et al (2010)^117^ | | CS (population-based) | | Brazil  (LAC, UM) | | Urban | | 3,597 | | Common mental disorders (SRQ-20, score: ≥7) | Income | | Yes | | Higher prevalence of CMD associated with lower income (PR=1.94 (95%CI: 1.62-2.32), aPR:1.89 (95% CI: 1.44-2.48)) | | Positive | | – | | Medium (no response rate, sensitivity of economic measure) | | |  |  |  |
| **OTHER MENTAL DISORDERS** | | | | | | | | | | | | | | | | | | | | |  | | |  |  |  |
| *ALL AGES* | |  | |  | |  | |  | |  |  | |  | |  | |  | |  | |  | | |  |  |  |
| Ataguba et al (2011)* | | CS (population-based) | | South Africa (SSA, UM) | | Both | | – | | Emotional disabilities (self-reported) | SES | | Yes | | Prevalence of emotional disabilities was disproportionately concentrated among lower SES quintiles (p<0.05) | | Positive | | – | | Low | | |  |  |  |
| Ayazi et al (2014) | | CS (population-based) | | South Sudan (SSA, L) | | Rural | | 1,200 | | Anxiety disorders (general, PTSD, panic disorder, OCD, etc) (MINI) | SES | | Yes | | People with any anxiety disorders were more likely to belong to the lowest SES category (many models, e.g. Men: aOR=4.6, 95% CI: 2.1-10.4; women: aOR=3.5, 95%CI: 1.0-11.9) | | Positive | | – | | Low | | |  |  |  |
| Gawde et al (2013) | | CS (population-based) | | India  (SA, LM) | | Urban | | 600 | | Psychiatric disorders (Symptoms Checklist 90) | Income | | Yes | | No significant difference in prevalence of psychiatric disorders between people from the highest two and lowest two income groups (aOR=1.0, 95%CI: 0.6-1.8) | | Non-significant | | Positive | | Medium (insufficient power for analyses) | | |  |  |  |
| Husain et al (2014) | | CS (population-based) | | Pakistan  (SA, LM) | | Urban | | 880 | | Psychological distress (SRQ-20) | Income | | Yes | | Having a low income associated with higher SRQ score, significant in pooled (p=0.01) and for women only (p<0.001) | | Positive | | – | | Low | | |  |  |  |
| Liu et al (2015b) | | CS (population-based) | | China  (EA, LM) | | Both | | 1.9 million | | Schizophrenia (screening + CE) | Income | | Yes | | People from the lowest household income per capita group were more likely to experience schizophrenia compared to the highest (p<0.05) | | Positive | | – | | Low | | |  |  |  |
| Trani et al (2015a) | | CC (hospital – cases; population – controls) | | India  (SA, LM) | | Urban | | 1,033 | | Schizophrenia, affective disorders (CE based on ICD-10) | Assets  Income  PCE | | Unclear | | People with severe mental illness had fewer assets (p<0.0001) and personal (p<0.0001)/household (p=0.002) income than controls. Not significant for PCE | | Positive | | Positive | | Medium (lack/unclear adjusting) | | |  |  |  |
| Wang et al (2015a) | | CS (population-based) | | China  (EA, UM) | | Urban | | 16,866 | | Severe mental stress (Perceived Stress Scale) | Income | | Yes | | People from the highest income group were less likely to experience severe mental stress compared to people from the lowest income group (aOR=0.3, 95% CI: 0.2-0.4) | | Positive | | – | | Low | | |  |  |  |
| *CHILDREN* | |  | |  | |  | |  | |  |  | |  | |  | |  | |  | |  | | |  |  |  |
| Ali et al (2013)* | | Cohort (population-based) | | Pakistan (SA, L) | | Urban | | 420 | | Emotional development (Early Child Development Tool, by Aga Khan University) | Income | | Yes | | No significant difference in emotional development between children from household’s with father’s income below or above 3500 rupees/month | | Non-significant | | – | | Medium (low response rate, high loss to follow-up) | | |  |  |  |
| Anselmi et al (2012) | | Cohort (hospital-based) | | Brazil | | Urban | |  | | Conduct, emotional or attention/hyperactivity problems (Strengths and Difficulties Questionnaire score, parent-reported) | Income | | Yes | | Prevalence of conduct, emotional and attentional/hyperactivity problems were higher in adolescents from families consistently in the lowest tertile of income compared to adolescents from the highest tertile. | | Positive | | – | | Low (hospital-based birth cohort) | | |  |  |  |
| Escueta et al (2014)* | | CC (population-based) | | Mix (5 countries) | | Both | | 1,780 | | Emotional difficulties (KABC-II) | Assets | | Yes | | Emotional difficulties score increased with increasing wealth (p<0.01) | | Positive | | – | | Low | | |  |  |  |
| Fortes et al (2016) | | CS (school-based) | | Brazil  (LAC, UM) | | Urban | | 1,338 | | DSM-5 learning disorders (Brazilian Academic Performance  Test; exclusion of ID) | SES | | Yes | | Learning disorders were more prevalent in the two lowest SES groups compared to the two highest (aOR=2.8, 95% CI: 1.0-8.2) | | Positive | | – | | Medium (school-based) | | |  |  |  |
| Petresco et al (2014) | | Cohort (hospital-based) | | Brazil  (LAC, UM) | | Urban | | 3,585 | | Psychiatric disorders | SES | | No | | Children born into lowest SES quintile were more likely to develop psychiatric disorders compared to children born in the highest SES quintile (p<0.001) | | Positive | | – | | Medium (no adjusting, unclear response rate) | | |  |  |  |
| Shams et al (2011) | | CS (high school students) | | Iran  (ME, UM) | | Rural | | 909 | | Obsessive compulsive disorder (Maudsley Obsessional-Compulsive Inventory and SCL-90-R) | Income | | No | | No significant association between level of income and prevalence of OCD, although OCD was more prevalent in the poorest income group compared to highest income group (OR= 2.78 (95% CI: 1.04-7.50)) | | Positive | | – | | Medium (no adjusting, response rate unclear, analysis unclear) | | |  |  |  |
| *ADULTS* | |  | |  | |  | |  | |  |  | |  | |  | |  | |  | |  | | |  |  |  |
| Awas et al (1999) | | CS (population-based) | | Ethiopia (SSA, L) | | Rural | | 501 | | Mental disorders (mood disorders, phobic disorders, other anxiety disorders, somatoform disorder) (CIDI) | Income | | Yes | | Prevalence of mental disorders was higher in the low income group compared to the medium and high income groups. This difference was only significant for mood disorders in low vs medium income groups. | | Positive | | – | | Medium (validity of economic measure) | | |  |  |  |
| Blue (2000) | | CS (population-based) | | Brazil  (LAC, UM) | | Urban | | 1,739 | | Psychiatric morbidity (Questionnaire for Adult Psychiatric Morbidity) | Income | | Yes | | Prevalence of psychiatric disability was higher in adults from families in the lowest income group compared to those in the highest (OR=2.34 (95% CI: 1.71-3.20), aOR=1.49 (95%CI: 1.0-2.2)). | | Positive | | – | | Low (no response rate) | | |  |  |  |
| Brown et al (2013) | | Pre-post (population-based) | | Vietnam (EA, L) | | Both | | 798 | | Psychiatric symptoms (SRQ-20) | Assets | | Yes | | Psychiatric symptoms increased with decreasing asset ownership (p<0.05) | | Positive | | – | | Low | | |  |  |  |
| Islam et al (2003) | | CS (population-based) | | Bangladesh  (SA, L) | | Urban | | 149 | | Psychiatric disorders (SRQ, CE) | PCE | | Yes | | Prevalence of psychiatric disorders increased significantly with higher per capita expenditure (ap<0.001) | | Negative | | – | | Low | | |  |  |  |
| Kawakami et al (2012) | | CS (population-based) | | 11 countries | | Both | | 37,741 | | Early onset (before individual completed education) mental disorders (CIDI, WMHS) | Income | | Yes | | Early onset mental disorders associated with low current household income significant in middle but not low income countries | | Positive, mixed significance | | Positive | | Medium (reliability of economic measure, high non-response to income questions) | | |  |  |  |
| Levinson et al (2010) | | CS (population-based) | | 9 countries | | Both | | 21,104 | | Serious mental illness (CIDI, serious = score in “severe range” on Sheehan Disability Scales or attempting suicide) | Income | | Yes | | Proportion of respondents with low and low-average earnings significantly higher among those with compared to without serious mental illness (p<0.001). Respondents with serious mental illness earned 33% less than median earnings (p<0.05) | | Positive | | – | | Medium (reliability of economic measure) | | |  |  |  |
| Li et al (2012) | | CS (population-based) | | China  (EA, LM) | | Both | | 1.8 million | | Psychiatric disability (CE, ICD-10 for diagnosis, WHO-DAS11 severity) | Income | | Yes | | People with psychiatric depression more likely to be living below poverty line (aOR= 2.25(95% CI: 2.15–2.35) | | Positive | | – | | Low (no response rate) | | |  |  |  |
| Li et al (2015a) | | CS (population-based) | | China  (EA, LM) | | Both | | 2.5 million | | Mood disorder (WHO-ICF, CE) | Income | | No | | People living below the national average for family income per capita were more likely than those living at or above the average to have a mood disorder (p=0.001) | | Positive | | – | | Medium (no adjusting) | | |  |  |  |
| Medina-Mora et al (2005) | | CS (population-based) | | Mexico  (LAC, UM) | | Urban | | 5,826 | | Psychiatric disorders, 12 month prevalence (CIDI, any DSM-IV disorder) | Income | | Yes | | People from low OR=2.7 (95% CI: 1.3-5.4) and low-average (aOR 2.0, 95% CI 1.0-4.0) incomes more likely to report severe disorder. No significant difference for other specific disorders (mood, anxiety, impulse-control or substance abuse) | | Positive | | – | | Medium (unclear economic measure, slightly low response rate) | | |  |  |  |
| Mokhtari et al (2013) | | CS (university students) | | Iran  (ME, UM) | | Urban | | 1,572 | | Mental health problems (GHQ-28) | Income | | No | | Poorer GHQ scores among lower income groups (p<0.05) | | Positive | | – | | Medium (no adjusting, school-based) | | |  |  |  |
| Myer et al (2008) | | CS (population-based) | | South Africa  (SSA, LM) | | Both | | 4,351 | | Psychological distress in past 30 days (K-10) | Income Assets SES | | Yes | | Prevalence of psychological distress significantly associated with SES (p<0.001). Prevalence higher among individuals in poorest income, asset and SES groupings compared to those in richest. | | Positive | | Positive | | Low | | |  |  |  |
| Nguyen et al (2016) | | CS (population-based) | | India, Vietnam, Ethiopia | | Both | | 5,647 | | Clinically significant maternal distress (SRQ-20) | SES | | Yes | | Mothers in the poorest SES group were more likely to have maternal distress compared to the highest SES group in Indian and Vietnam. There was no significant difference in Ethiopia. | | Positive | | Positive | | Low | | |  |  |  |
| Norris et al (2003) | | CS (population-based) | | Mexico  (LAC, UM) | | Urban | | 2,509 | | Post-traumatic stress disorder (Module K of CIDI) | SES | | Yes | | Prevalence of PTSD increased with decreasing SES (p<0.001) | | Positive | | – | | Low | | |  |  |  |
| Ou et al (2015)* | | CC (school-based) | | China  (EA, UM) | | Both | | 1,301 | | Autistic spectrum disorder (self-report of diagnosis, CE using DSM-4) | Income | | Yes | | There was a significant difference between households with and without a child with autism (p<0.001) | | Positive | | – | | Medium (likely selection bias, controls not well matched on gender) | | |  |  |  |
| Santos et al (2014) | | Cohort (clinic based) | | Brazil  (LAC, UM) | | Urban | | 3,581 | | Antenatal and postnatal mood symptoms (SRQ-20) | Income | | No | | Higher prevalence of mood symptoms in women during pregnancy and postpartum from families with lower incomes (p trend <0.001) | | Positive | | – | | Medium (not adjusted, hospital-based) | | |  |  |  |
| Sharifi et al (2015) | | CS (population-based) | | Iran  (ME, UM) | | Both | | 7,886 | | Psychiatric disorders (screen with CIDI 2, SCID-1 for CE) | Assets | | Yes | | People with higher SES have lower likelihood of having a psychiatric disorder (high to low: aOR=0.64 (0.52-0.78) | | Positive | | Positive | | Low | | |  |  |  |
| Wang et al (2015d) | | CS  (population-based) | | China  (EA, LM) | | Both | | 1.7 million | | Mental disability (CE with ICD-10) | SES | | Yes | | Mental disability was significantly more prevalent among lower wealth groups (p trend<0.01; aOR highest to lowest: 0.15, 95%CI: 0.13-0.16) | | Positive | | – | | Low | | |  |  |  |
| Xiang et al (2008) | | CS (population-based) | | China  (EA, LM) | | Urban | | 5,926 | | Schizophrenia (CIDI, ICD-10) | Income | | Yes | | Higher prevalence of schizophrenia among poorest group compared to wealthiest: OR = 14.29 (95% CI: 1.92-111.1); aOR = 19.23 (95% CI: 1.79-200) | | Positive | | Non-significant | | Medium (lacks power, confidence intervals very broad) | | |  |  |  |
| *OLDER ADULTS* | | | | | | | | | |  |  | |  | |  | |  | |  | |  | | |  |  |  |
| Soares et al (2015) | | CS (population-based) | | Brazil  (LAC, UM) | | Urban | | 1,125 | | Psychotic symptoms (Cambridge Mental Disorders of the Elderly Examination) | SES | | Yes | | People belonging to the lowest two social classes were more likely to experience psychotic symptoms compared to people in the top 3 classes (aOR=2.1, 95% CI: 1.2-3.7) | | Positive | | – | | Medium (validity of disability measure, slightly low response rate) | | |  |  |  |
| Study design abbreviations: CC=case control, CS=cross-sectional; Means of assessment abbreviations: CE=clinical evaluation, CERAD=Clinical and Neuropsychology Assessment, CIDI=Composite International Diagnostic Interview, DSM: Diagnostic and Statistical Manual, GDS: Geriatric Depression Scale, GHQ-20: General Health Questionnaire, GMS-AGECAT: Geriatric Mental State-Automated Geriatric Examination for Computer Assisted Taxonomy, ICD: International Classification of Disease, MMSE: Mini Mental State Examination, SCL-90-R: Symptom Checklist-90-Revised, SRQ: Self-Reporting Questionnaire, WMHS: World Mental Health Survey, WHODAS: WHO Disability Assessment Schedule; Economic measure abbreviation: SES=socioeconomic status, PCE=per capita expenditures; Overview of results abbreviations: OR=odds ratio, aOR=adjusted odds ratio, CI=confidence interval; CMD=common mental disorders; *study is repeated in more than one category (results have been disaggregated by disability type) | | | | | | | | | | | | | | | | | | | | |  | | |  |  |  |
| ***Table 6:*** *Summary of studies examining mental disorders* | | | | | | | | | | | | | | | | | | | | |  | | |  |  |  |

**PRISMA Checklist**

| **Section/topic** | **#** | **Checklist item** | **Reported on page #** |
| --- | --- | --- | --- |
| **TITLE** | | |  |
| Title | 1 | Identify the report as a systematic review, meta-analysis, or both. | 1 |
| **ABSTRACT** | | |  |
| Structured summary | 2 | Provide a structured summary including, as applicable: background; objectives; data sources; study eligibility criteria, participants, and interventions; study appraisal and synthesis methods; results; limitations; conclusions and implications of key findings; systematic review registration number. | 1-2 |
| **INTRODUCTION** | | |  |
| Rationale | 3 | Describe the rationale for the review in the context of what is already known. | 3 |
| Objectives | 4 | Provide an explicit statement of questions being addressed with reference to participants, interventions, comparisons, outcomes, and study design (PICOS). | 4 |
| **METHODS** | | |  |
| Protocol and registration | 5 | Indicate if a review protocol exists, if and where it can be accessed (e.g., Web address), and, if available, provide registration information including registration number. | n/a |
| Eligibility criteria | 6 | Specify study characteristics (e.g., PICOS, length of follow-up) and report characteristics (e.g., years considered, language, publication status) used as criteria for eligibility, giving rationale. | 4-5 |
| Information sources | 7 | Describe all information sources (e.g., databases with dates of coverage, contact with study authors to identify additional studies) in the search and date last searched. | 4 |
| Search | 8 | Present full electronic search strategy for at least one database, including any limits used, such that it could be repeated. | Supporting information |
| Study selection | 9 | State the process for selecting studies (i.e., screening, eligibility, included in systematic review, and, if applicable, included in the meta-analysis). | 5-6 |
| Data collection process | 10 | Describe method of data extraction from reports (e.g., piloted forms, independently, in duplicate) and any processes for obtaining and confirming data from investigators. | 6 |
| Data items | 11 | List and define all variables for which data were sought (e.g., PICOS, funding sources) and any assumptions and simplifications made. | 6-7 |
| Risk of bias in individual studies | 12 | Describe methods used for assessing risk of bias of individual studies (including specification of whether this was done at the study or outcome level), and how this information is to be used in any data synthesis. | 5-6 |
| Summary measures | 13 | State the principal summary measures (e.g., risk ratio, difference in means). | 6-7 |
| Synthesis of results | 14 | Describe the methods of handling data and combining results of studies, if done, including measures of consistency (e.g., I^2^) for each meta-analysis. | n/a |
| Risk of bias across studies | 15 | Specify any assessment of risk of bias that may affect the cumulative evidence (e.g., publication bias, selective reporting within studies). | n/a |
| Additional analyses | 16 | Describe methods of additional analyses (e.g., sensitivity or subgroup analyses, meta-regression), if done, indicating which were pre-specified. | n/a |
| **RESULTS** | | |  |
| Study selection | 17 | Give numbers of studies screened, assessed for eligibility, and included in the review, with reasons for exclusions at each stage, ideally with a flow diagram. | 7, fig 1 |
| Study characteristics | 18 | For each study, present characteristics for which data were extracted (e.g., study size, PICOS, follow-up period) and provide the citations. | Supporting information, 8 |
| Risk of bias within studies | 19 | Present data on risk of bias of each study and, if available, any outcome level assessment (see item 12). |  |
| Results of individual studies | 20 | For all outcomes considered (benefits or harms), present, for each study: (a) simple summary data for each intervention group (b) effect estimates and confidence intervals, ideally with a forest plot. | Supporting information, 10, 11 |
| Synthesis of results | 21 | Present results of each meta-analysis done, including confidence intervals and measures of consistency. | n/a |
| Risk of bias across studies | 22 | Present results of any assessment of risk of bias across studies (see Item 15). | n/a |
| Additional analysis | 23 | Give results of additional analyses, if done (e.g., sensitivity or subgroup analyses, meta-regression [see Item 16]). | n/a |
| **DISCUSSION** | | |  |
| Summary of evidence | 24 | Summarize the main findings including the strength of evidence for each main outcome; consider their relevance to key groups (e.g., healthcare providers, users, and policy makers). | 13-16 |
| Limitations | 25 | Discuss limitations at study and outcome level (e.g., risk of bias), and at review-level (e.g., incomplete retrieval of identified research, reporting bias). | 15-16 |
| Conclusions | 26 | Provide a general interpretation of the results in the context of other evidence, and implications for future research. | 15-16 |
| **FUNDING** | | |  |
| Funding | 27 | Describe sources of funding for the systematic review and other support (e.g., supply of data); role of funders for the systematic review. |  |
