# Supplementary material for: Poverty and disability in low- and middle-income countries: A systematic review
Source: PLoS One. 2017 Dec 21;12(12):e0189996. doi: 10.1371/journal.pone.0189996 (PMC5739437; doi:10.1371/journal.pone.0189996)
Supplement: S2 File — (DOCX) [file pone.0189996.s004.docx]

**References of included studies**

1. Abas MA, Broadhead JC. Depression and anxiety among women in an urban setting in Zimbabwe. Psychological medicine. 1997;27(1):59-71.

2. Ali NS, Mahmud S, Khan A, Ali BS. Impact of postpartum anxiety and depression on child's mental development from two peri-urban communities of Karachi, Pakistan: a quasi-experimental study. Bmc Psychiatry. 2013;13. doi: 10.1186/1471-244x-13-274. PubMed PMID: WOS:000326296100001.

3. Anselmi L, Barros FC, Minten GC, Gigante DP, Horta BL, Victora CG. [Prevalence and early determinants of common mental disorders in the 1982 birth cohort, Pelotas, Southern Brazil]. Rev Saude Publica. 2008;42 Suppl 2:26-33. Epub 2009/01/30. PubMed PMID: 19142342; PubMed Central PMCID: PMCPMC2667238.

4. Anselmi L, Menezes AMB, Hallal PC, Wehrmeister F, Goncalves H, Barros FC, et al. Socioeconomic changes and adolescent psychopathology in a Brazilian birth cohort study. (Special Issue: Health from birth to adolescence: findings from the 1993 Pelotas, Brazil, Birth Cohort Study.). Journal of Adolescent Health. 2012;51(6, Suppl):S5-S10. PubMed PMID: 20123417530.

5. Arguvanli S, Akn S, Safak ED, Mucuk S, Ozturk A, Mazcoglu MM, et al. Prevalence of cognitive impairment and related risk factors in community-dwelling elderly in Kayseri, Turkey. (Special Issue: Alzheimer disease: form bench to bedside.). Turkish Journal of Medical Sciences. 2015;45(5):1167-72. PubMed PMID: 20153369240.

6. Arokiasamy P, Uttamacharya U, Jain K, Biritwum RB, Yawson AE, Fan W, et al. The impact of multimorbidity on adult physical and mental health in low- and middle-income countries: what does the study on global ageing and adult health (SAGE) reveal? BMC Medicine. 2015;13(1):1-16. doi: 10.1186/s12916-015-0402-8. PubMed PMID: 109221568.

7. Ataguba JE, Akazili J, McIntyre D. Socioeconomic-related health inequality in South Africa: Evidence from General Household Surveys. International Journal for Equity in Health. 2011;10(48). PubMed PMID: 2011666120.

8. Awas M, Kebede D, Alem A. Major mental disorders in Butajira, southern Ethiopia. Acta Psychiatrica Scandinavica, Supplementum. 1999;397:56-64. PubMed PMID: 10470356.

9. Ayazi T, Lien L, Eide A, Swartz L, Hauff E. Association between exposure to traumatic events and anxiety disorders in a post-conflict setting: a cross-sectional community study in South Sudan. BMC Psychiatry. 2014;14(1):1-22. doi: 10.1186/1471-244X-14-6. PubMed PMID: 94426987.

10. Ball HA, Siribaddana SH, Kovas Y, Glozier N, McGuffin P, Sumathipala A, et al. Epidemiology and symptomatology of depression in Sri Lanka: a cross-sectional population-based survey in Colombo District. Journal of Affective Disorders. 2010;123(1/3):188-96. PubMed PMID: 20103161940.

11. Basu S, King AC. Disability and Chronic Disease Among Older Adults in India: Detecting Vulnerable Populations Through the WHO SAGE Study. American Journal of Epidemiology. 2013;178(11):1620-8. PubMed PMID: 92712593.

12. Béria JU, Raymann BCW, Gigante LP, Figueiredo ACL, Jotz G, Roithman R, et al. Hearing impairment and socioeconomic factors: a population-based survey of an urban locality in southern Brazil. Deficiencia auditiva y factores socioeconómicos: estudio de base poblacional en una localidad urbana del sur de Brasil. 2007;21(6):381-7. PubMed PMID: 26609456.

13. Beydoun MA, Popkin BM. The impact of socio-economic factors on functional status decline among community-dwelling older adults in China. Social Science & Medicine. 2005;60(9):2045-57. PubMed PMID: 15743653.

14. Blay SL, Andreoli SB, Fillenbaum GG, Gastal FL. Depression morbidity in later life: prevalence and correlates in a developing country. American Journal of Geriatric Psychiatry. 2007;15(9):790-9. PubMed PMID: 17698602.

15. Blay SL, Fillenbaum GG, Andreoli SB, Gastal FL. Prevalence and concomitants of arthritis in the elderly in Rio Grande do Sul, Brazil. PLoS ONE [Electronic Resource]. 2012;7(9):e45418. PubMed PMID: 23028995; PubMed Central PMCID: PMCPMC3460974.

16. Blue I. Individual and contextual effects on mental health status in Sao Paulo, Brazil. Revista Brasileira de Psiquiatria. 2000;22(3):116-23. doi: 10.1590/s1516-44462000000300004. PubMed PMID: BCI:BCI200000509027.

17. Brown R, Trapp S, Berenz E, Bigdeli T, Acierno R, Tran T, et al. Pre-typhoon socioeconomic status factors predict post-typhoon psychiatric symptoms in a Vietnamese sample. Social Psychiatry & Psychiatric Epidemiology. 2013;48(11):1721-7. doi: 10.1007/s00127-013-0684-0. PubMed PMID: 90794012.

18. Chen L, Wang L, Qiu XH, Yang XX, Qiao ZX, Yang YJ, et al. Depression among Chinese university students: prevalence and socio-demographic correlates. PLoS One. 2013;8(3):e58379. Epub 2013/03/22. doi: 10.1371/journal.pone.0058379. PubMed PMID: 23516468; PubMed Central PMCID: PMCPMC3596366.

19. Chen R, Hu Z, Wei L, Ma Y, Liu Z, Copeland JR. Incident dementia in a defined older Chinese population. PLoS ONE [Electronic Resource]. 2011;6(9):e24817. PubMed PMID: 21966372; PubMed Central PMCID: PMCPMC3179466.

20. Chen R, Ma Y, Wilson K, Hu Z, Sallah D, Wang J, et al. A multicentre community-based study of dementia cases and subcases in older people in China-the GMS-AGECAT prevalence and socio-economic correlates. International Journal of Geriatric Psychiatry. 2012;27(7):692-702. doi: 10.1002/gps.2767. PubMed PMID: WOS:000305082000004.

21. Chen RL, Wei L, Hu Z, Qin X, Copeland JRM, Hemingway H. Depression in older people in rural China. Archives of Internal Medicine. 2005;165(17):2019-25. doi: 10.1001/archinte.165.17.2019. PubMed PMID: WOS:000232112800012.

22. Cockburn N, Steven D, Lecuona K, Joubert F, Rogers G, Cook C, et al. Prevalence, causes and socio-economic determinants of vision loss in Cape Town, South Africa. PLoS ONE. 2012;7(2). PubMed PMID: 20123110590.

23. Coelho FM, Pinheiro RT, Horta BL, Magalhaes PV, Garcias CM, Silva CV. Common mental disorders and chronic non-communicable diseases in adults: a population-based study. Cadernos de saude publica. 2009;25(1):59-67. Epub 2009/01/31. PubMed PMID: 19180287.

24. Cordeiro de Andrade KR, Silva MT, Galvao TF, Pereira MG. Functional disability of adults in Brazil: prevalence and associated factors. Revista de Saude Publica. 2015;49. PubMed PMID: 26759965; PubMed Central PMCID: PMCPMC4687823.

25. Dandona L, Dandona R, Marmamula S, Pyda G, Kovai V, Prasad MN, et al. Blindness in the Indian state of Andhra Pradesh. Investigative Ophthalmology & Visual Science. 2001;42(5):908-16. PubMed PMID: 20023072958.

26. Dandona R, Dandona L, Srinivas M, Giridhar P, Prasad MN, Vilas K, et al. Moderate visual impairment in India: the Andhra Pradesh Eye Disease Study. British Journal of Ophthalmology. 2002;86(4):373-7. PubMed PMID: 11914201; PubMed Central PMCID: PMCPMC1771093.

27. Dang HM, Weiss B, Trung LT. Functional impairment and mental health functioning among Vietnamese children. Social Psychiatry & Psychiatric Epidemiology. 2016;51(1):39-47. PubMed PMID: 26315942; PubMed Central PMCID: PMCNIHMS719217 [Available on 01/01/17]

PMC4724307 [Available on 01/01/17].

28. Danquah L, Polack S, Brus A, Mactaggart I, Houdon CP, Senia P, et al. Disability in post-earthquake Haiti: prevalence and inequality in access to services. Disability and rehabilitation. 2015;37(12):1082-9. Epub 2014/09/06. doi: 10.1016/j.socscimed.2014.08.021

10.3109/09638288.2014.956186. PubMed PMID: 25178862.

29. Dasgupta A, Ray D, Roy S, Sarkar T, Ghosal A, Das A, et al. Depression among the Geriatric Population is a Matter of Concern: A Community Based Study in a Rural Area of West Bengal. Nepal Journal of Epidemiology. 2013;3(4):282-7. PubMed PMID: 94601776.

30. Dorsi E, Xavier A, Schneider I, Corseuil M, Ramos L. Factors associated with cognitive impairment in older adults: A population based survey, South Brazil. Alzheimer's and Dementia. 2011;1):S370. PubMed PMID: 70501771.

31. Emamian MH, Zeraati H, Majdzadeh R, Shariati M, Hashemi H, Fotouhi A. The gap of visual impairment between economic groups in Shahroud, Iran: a Blinder-Oaxaca decomposition. American Journal of Epidemiology. 2011;173(12):1463-7. PubMed PMID: 21540323.

32. Emamian MH, Zeraati H, Majdzadeh R, Shariati M, Hashemi H, Jafarzadehpur E, et al. Economic inequality in presenting near vision acuity in a middle-aged population: a Blinder-Oaxaca decomposition. British Journal of Ophthalmology. 2013;97(9):1100-3. PubMed PMID: 20133317510.

33. Ergin I, Kunst AE. Regional inequalities in self-rated health and disability in younger and older generations in Turkey: the contribution of wealth and education. BMC Public Health. 2015;15(987). PubMed PMID: 20153353628.

34. Escueta M, Whetten K, Ostermann J, O'Donnell K. Adverse childhood experiences, psychosocial well-being and cognitive development among orphans and abandoned children in five low income countries. BMC International Health & Human Rights. 2014;14(1):1-13. doi: 10.1186/1472-698X-14-6. PubMed PMID: 96096483.

35. Falkingham JC, Chepngeno-Langat G, Kyobutungi C, Ezeh A, Evandrou M. Does socioeconomic inequality in health persist among older people living in resource-poor urban slums? Journal of Urban Health. 2011;88 Suppl 2:S381-400. PubMed PMID: 21431465.

36. Fei M, Qu YC, Wang T, Yin J, Bai JX, Ding QH. Prevalence and distribution of cognitive impairment no dementia (CIND) among the aged population and the analysis of socio-demographic characteristics: the community-based cross-sectional study. Alzheimer Disease & Associated Disorders. 2009;23(2):130-8. PubMed PMID: 19474570.

37. Fernandez-Nino JA, Manrique-Espinoza BS, Bojorquez-Chapela I, Salinas-Rodriguez A. Income inequality, socioeconomic deprivation and depressive symptoms among older adults in Mexico. PLoS One. 2014;9(9):e108127. Epub 2014/09/25. doi: 10.1371/journal.pone.0108127. PubMed PMID: 25250620; PubMed Central PMCID: PMCPmc4176015.

38. Fillenbaum GG, Blay SL, Andreoli SB, Gastal FL. Prevalence and Correlates of Functional Status in an Older Community-Representative Sample in Brazil. Journal of aging and health. 2010;22(3):362-83. doi: 10.1177/0898264309359307. PubMed PMID: WOS:000275441800006.

39. Filmer D. Disability, poverty, and schooling in developing countries: Results from 14 household surveys. World Bank Economic Review. 2008;22(1):141-63. doi: 10.1093/wber/lhm021. PubMed PMID: WOS:000253859500007.

40. Fortes I, Paula C, Oliveira M, Bordin I, de Jesus Mari J, Rohde L. A cross-sectional study to assess the prevalence of DSM-5 specific learning disorders in representative school samples from the second to sixth grade in Brazil. European Child & Adolescent Psychiatry. 2016;25(2):195-207. doi: 10.1007/s00787-015-0708-2. PubMed PMID: 112692762.

41. Freeman EE, Roy-Gagnon MH, Samson E, Haddad S, Aubin MJ, Vela C, et al. The Global Burden of Visual Difficulty in Low, Middle, and High Income Countries. PLoS ONE. 2013;8 (5) (no pagination)(e63315). PubMed PMID: 2013300921.

42. Gawde N, Nasirabadi M, Shah N, Nagaonkar S. Psychiatric morbidity in an urban slum of Mumbai: cross sectional study. Asian journal of psychiatry. 2013;6(6):478-82. Epub 2013/12/07. doi: 10.1016/j.ajp.2013.05.005. PubMed PMID: 24309857.

43. Graham E, Jordan LP, Yeoh BS. Parental migration and the mental health of those who stay behind to care for children in South-East Asia. Social science & medicine (1982). 2015;132:225-35. Epub 2014/12/04. doi: 10.1016/j.socscimed.2014.10.060. PubMed PMID: 25464878; PubMed Central PMCID: PMCPmc4405005.

44. Guerra M, Ferri CP, Sosa AL, Salas A, Gaona C, Gonzales V, et al. Late-life depression in Peru, Mexico and Venezuela: The 10/66 population-based study. British Journal of Psychiatry. 2009;195(6):510-5. PubMed PMID: 2009647213.

45. Guerra RO, Alvarado BE, Zunzunegui MV. Life course, gender and ethnic inequalities in functional disability in a Brazilian urban elderly population. Aging-Clinical & Experimental Research. 2008;20(1):53-61. PubMed PMID: 18283229.

46. Guo S, Tian D, Wang X, Xiao Y, He H, Qu Z, et al. Protective Effects of Social Support Content and Support Source on Depression and Its Prevalence 6 Months after Wenchuan Earthquake. Stress & Health: Journal of the International Society for the Investigation of Stress. 2015;31(5):382-92. doi: 10.1002/smi.2563. PubMed PMID: 111408147.

47. Gureje O, Kola L, Afolabi E. Epidemiology of major depressive disorder in elderly Nigerians in the Ibadan Study of Ageing: a community-based survey. Lancet. 2007;370(9591):957-64. Epub 2007/09/18. doi: 10.1016/s0140-6736(07)61446-9. PubMed PMID: 17869636; PubMed Central PMCID: PMCPMC2880490.

48. Gureje O, Ogunniyi A, Kola L, Afolabi E. Functional disability in elderly Nigerians: Results from the Ibadan Study of Aging. Journal of the American Geriatrics Society. 2006;54(11):1784-9. PubMed PMID: 17087709; PubMed Central PMCID: PMCPMC2820715

UKMS28650.

49. Habtamu E, Wondie T, Aweke S, Tadesse Z, Zerihun M, Zewdie Z, et al. Trachoma and Relative Poverty: A Case-Control Study. PLoS Neglected Tropical Diseases [electronic resource]. 2015;9(11):e0004228. PubMed PMID: 26600211; PubMed Central PMCID: PMCPMC4657919.

50. Halpern R, Barros AJD, Matijasevich A, Santos IS, Victora CG, Barros FC. Developmental status at age 12 months according to birth weight and family income: A comparison of two Brazilian birth cohorts. Cadernos de Saude Publica. 2008;24(SUPPL.3):S444-S50. PubMed PMID: 18797720.

51. Hanandita W, Tampubolon G. Does poverty reduce mental health? An instrumental variable analysis. Social science & medicine (1982). 2014;113:59-67. Epub 2014/05/20. doi: 10.1016/j.socscimed.2014.05.005. PubMed PMID: 24836844.

52. Herrera E, Caramelli P, Silveira ASB, Nitrini R. Epidemiologic survey of dementia in a community-dwelling Brazilian population. Alzheimer Disease & Associated Disorders. 2002;16(2):103-8. doi: 10.1097/01.WAD.0000020202.50697.df. PubMed PMID: WOS:000176910900006.

53. Ho VH, Schwab IR. Social economic development in the prevention of global blindness. British Journal of Ophthalmology. 2001;85(6):653-7. PubMed PMID: 11371481; PubMed Central PMCID: PMCPMC1724021.

54. Hoogeveen JG. Measuring welfare for small but vulnerable groups: Poverty and disability in Uganda. Journal of African Economies. 2005;14(4):603-31.

55. Hosseinpoor AR, Bergen N, Mendis S, Harper S, Verdes E, Kunst A, et al. Socioeconomic inequality in the prevalence of noncommunicable diseases in low- and middle-income countries: results from the World Health Survey. BMC Public Health. 2012;12:474. PubMed PMID: 22726343; PubMed Central PMCID: PMCPMC3490890.

56. Hosseinpoor AR, Williams JAS, Gautam J, Posarac A, Officer A, Verdes E, et al. Socioeconomic inequality in disability among adults: a multicountry study using the World Health Survey. American Journal of Public Health. 2013;103(7):1278-86. PubMed PMID: 20133239665.

57. Husain N, Nasim C, Farhat J, Tomenson B, Ishaq S, Ilyas M, et al. Prevalence and risk factors for psychological distress and functional disability in urban Pakistan. WHO South East Asia Journal of Public Health. 2014;3(2):144-53. PubMed PMID: 20153244515.

58. Ibrahim AK, Kelly SJ, Glazebrook C. Analysis of an Egyptian study on the socioeconomic distribution of depressive symptoms among undergraduates. Social psychiatry and psychiatric epidemiology. 2012;47(6):927-37. Epub 2011/06/01. doi: 10.1007/s00127-011-0400-x. PubMed PMID: 21626055.

59. Islam M, Ali M, Ferroni P, Underwood P, FaruqAlam M. Prevalence of psychiatric disorders in an urban community in Bangladesh. General hospital psychiatry. 2003;25(5):353-7. PubMed PMID: 64591385; 15161304.

60. Jenkins R, Othieno C, Ongeri L, Sifuna P, Ongecha M, Kingora J, et al. Common mental disorder in Nyanza province, Kenya in 2013 and its associated risk factors -an assessment of change since 2004, using a repeat household survey in a demographic surveillance site. BMC Psychiatry. 2015;15:1-12. doi: 10.1186/s12888-015-0693-5. PubMed PMID: 111548766.

61. Jiang NM, Tofail F, Scharf RJ, Moonah SN, Taniuchi M, Ma JZ, et al. Febrile illness and pro-inflammatory cytokines in the first year of life predict impaired child development in Bangladeshi infants living in poverty. American Journal of Tropical Medicine and Hygiene. 2013;1):141. PubMed PMID: 71312296.

62. Kawakami N, Abdulghani EA, Alonso J, Bromet EJ, Bruffaerts R, Caldas-de-almeida JM, et al. Early-Life Mental Disorders and Adult Household Income in the World Mental Health Surveys. Biological psychiatry (1969). 2012;72(3):228-37. PubMed PMID: 1268687365; 26185901.

63. Kawakatsu Y, Kaneko S, Karama M, Honda S. Prevalence and risk factors of neurological impairment among children aged 6-9 years: from population based cross sectional study in western Kenya. BMC Pediatrics. 2012;12:186. PubMed PMID: 23206271; PubMed Central PMCID: PMCPMC3519515.

64. Keskinoglu P, Giray H, Pıcakcıefe M, Bilgic N, Ucku R. The prevalence and risk factors of dementia in the elderly population in a low socio-economic region of Izmir, Turkey. Archives of Gerontology & Geriatrics. 2006;43(1):93-100. doi: 10.1016/j.archger.2005.09.006. PubMed PMID: 21187054.

65. Kilzieh N, Rastam S, Ward KD, Maziak W. Gender, depression and physical impairment: an epidemiologic perspective from Aleppo, Syria. Social Psychiatry & Psychiatric Epidemiology. 2010;45(6):595-602. PubMed PMID: 20195569; PubMed Central PMCID: PMCNIHMS190344

PMC2874618.

66. Kulkarni RS, Shinde RL. Depression and Its Associated Factors in Older Indians: A Study Based on Study of Global Aging and Adult Health (SAGE)-2007. Journal of aging and health. 2015;27(4):622-49. Epub 2014/11/06. doi: 10.1177/0898264314556617. PubMed PMID: 25370713.

67. Kumar R, Aggarwal AK, Kaur M, Iyengar SD. Factors influencing psychosocial development of preschool children in a rural area of Haryana, India. Journal of Tropical Pediatrics. 1997;43(6):324-9. doi: 10.1093/tropej/43.6.324. PubMed PMID: WOS:000071747500003.

68. Kumar R, Anupama B, Roli B, Agarwal GG. Prevalence and risk factors for neurological disorders in children aged 6 months to 2 years in northern India. Developmental Medicine & Child Neurology. 2013;55(4):348-56. PubMed PMID: 20133140788.

69. Kuper H, Monteath-van Dok A, Wing K, Danquah L, Evans J, Zuurmond M, et al. The impact of disability on the lives of children; cross-sectional data including 8,900 children with disabilities and 898,834 children without disabilities across 30 countries. PLoS One. 2014;9(9):e107300. Epub 2014/09/10. doi: 10.1371/journal.pone.0107300. PubMed PMID: 25202999; PubMed Central PMCID: PMCPmc4159292.

70. Kuper H, Nyapera V, Evans J, Munyendo D, Zuurmond M, Frison S, et al. Malnutrition and childhood disability in Turkana, Kenya: Results from a case-control study. PloS one. 2015;10(12):e0144926.

71. Kuper H, Polack S, Foster A, Eusebio C, Mathenge W, Wadud Z. A case-control study to assess the relationship between poverty and visual impairment from cataract in Kenya, the Philippines, and Bangladesh. PLoS Medicine. 2008;5(12):1716-28. PubMed PMID: 2009021597.

72. Lei X, Sun X, Strauss J, Zhang P, Zhao Y. Depressive symptoms and SES among the mid-aged and elderly in China: Evidence from the China Health and Retirement Longitudinal Study national baseline. Social Science & Medicine. 2014;120:224-32. doi: 10.1016/j.socscimed.2014.09.028. PubMed PMID: WOS:000345180600026.

73. Levinson D, Lakoma MD, Petukhova M, Schoenbaum M, Zaslavsky AM, Angermeyer M, et al. Associations of serious mental illness with earnings: results from the WHO World Mental Health surveys. British journal of psychiatry (Print). 2010;197(AOU):114-21. PubMed PMID: 854107581; 23247855.

74. Li N, Du W, Zhang L, Chen G, Zheng X. Prevalence and functions of mental disability caused by mood disorders in China: A national sample. Journal of affective disorders. 2015a;180:10-3. Epub 2015/04/17. doi: 10.1016/j.jad.2015.03.016. PubMed PMID: 25879719.

75. Li N, Pang L, Chen G, Song X, Zhang J, Zheng X. Risk factors for depression in older adults in Beijing. Canadian journal of psychiatry Revue canadienne de psychiatrie. 2011;56(8):466-73. Epub 2011/09/01. PubMed PMID: 21878157.

76. Li N, Pang L, Du W, Chen G, Zheng X. Association between poverty and psychiatric disability among Chinese population aged 15–64 years. Psychiatry Research. 2012;200(2/3):917-20. doi: 10.1016/j.psychres.2012.05.026. PubMed PMID: 83869402.

77. Li N, Zhang L, Du W, Pang L, Guo C, Chen G, et al. Prevalence of dementia-associated disability among chinese older adults: Results from a national sample survey. American Journal of Geriatric Psychiatry. 2015b;23(3):320-5. PubMed PMID: 2015951904.

78. Lima MS, Beria JU, Tomasi E, Conceicao AT, Mari JJ. Stressful life events and minor psychiatric disorders: an estimate of the population attributable fraction in a Brazilian community-based study. The International Journal of Psychiatry in Medicine. 1996;26(2):211-22.

79. Lin T, Li N, Du W, Song X, Zheng X. Road traffic disability in China: prevalence and socio-demographic disparities. Journal of public health (Oxford, England). 2013. Epub 2013/02/07. doi: 10.1093/pubmed/fdt003. PubMed PMID: 23386326.

80. Liu J, Chi I, Chen G, Song X, Zheng X. Prevalence and correlates of functional disability in Chinese older adults. Geriatrics & gerontology international. 2009;9(3):253-61. PubMed PMID: 19702935.

81. Liu J, Yan F, Ma X, Guo HL, Tang YL, Rakofsky JJ, et al. Prevalence of major depressive disorder and socio-demographic correlates: Results of a representative household epidemiological survey in Beijing, China. Journal of affective disorders. 2015a;179:74-81. Epub 2015/04/08. doi: 10.1016/j.jad.2015.03.009. PubMed PMID: 25845752.

82. Liu T, Zhang L, Pang L, Li N, Chen G, Zheng X. Schizophrenia-related disability in China: prevalence, gender, and geographic location. Psychiatric services (Washington, DC). 2015b;66(3):249-57. Epub 2015/03/03. doi: 10.1176/appi.ps.201400032. PubMed PMID: 25727112.

83. Lopes MA, Hototian SR, Bustamante SEZ, Azevedo D, Tatsch M, Bazzarella MC, et al. Prevalence of cognitive and functional impairment in a community sample in Ribeirão Preto, Brazil. International Journal of Geriatric Psychiatry. 2007;22(8):770-6. doi: 10.1002/gps.1737. PubMed PMID: 25960263.

84. Loyalka P, Liu L, Chen G, Zheng X. The cost of disability in China. Demography. 2014;51(1):97-118. Epub 2014/01/05. doi: 10.1007/s13524-013-0272-7. PubMed PMID: 24385200.

85. Ludermir AB, Lewis G. Links between social class and common mental disorders in Northeast Brazil. Social psychiatry and psychiatric epidemiology. 2001;36(3):101-7. Epub 2001/07/24. PubMed PMID: 11465780.

86. Ma X, Xiang Y-T, Cai Z-J, Lu J-Y, Li S-R, Xiang Y-Q, et al. Generalized Anxiety Disorder in China: Prevalence, Sociodemographic Correlates, Comorbidity, and Suicide Attempts. Perspectives in Psychiatric Care. 2009;45(2):119-27. doi: 10.1111/j.1744-6163.2009.00212.x. PubMed PMID: WOS:000264891600006.

87. Malhotra R, Chan A, Ostbye T. Prevalence and correlates of clinically significant depressive symptoms among elderly people in Sri Lanka: findings from a national survey. International Psychogeriatrics. 2010;22(2):227-36. PubMed PMID: 19747421.

88. Marella M, Huq NL, Devine A, Baker SM, Quaiyum MA, Keeffe JE. Prevalence and correlates of disability in Bogra district of Bangladesh using the rapid assessment of disability survey. BMC Public Health. 2015;15(867). PubMed PMID: 20153324478.

89. Mathenge W, Bastawrous A, Foster A, Kuper H. The Nakuru posterior segment eye disease study: methods and prevalence of blindness and visual impairment in Nakuru, Kenya. Ophthalmology. 2012;119(10):2033-9.

90. Medina-Mora ME, Borges G, Lara C, Benjet C, Blanco J, Fleiz C, et al. Prevalence, service use, and demographic correlates of 12-month DSM-IV psychiatric disorders in Mexico: results from the Mexican National Comorbidity Survey. Psychological medicine. 2005;35(12):1773-83. Epub 2005/11/23. doi: 10.1017/s0033291705005672. PubMed PMID: 16300691.

91. Melzer D, Parahyba MI. Socio-demographic correlates of mobility disability in older Brazilians: results of the first national survey. Age & Ageing. 2004;33(3):253-9. PubMed PMID: 15082430.

92. Minh HV, Giang KB, Liem NT, Palmer M, Thao NP, Duong LB. Estimating the extra cost of living with disability in Vietnam. Global Public Health. 2015;10:S70-S9. doi: 10.1080/17441692.2014.971332. PubMed PMID: 100640127.

93. Minicuci N, Biritwum RB, Mensah G, Yawson AE, Naidoo N, Chatterji S, et al. Sociodemographic and socioeconomic patterns of chronic non-communicable disease among the older adult population in Ghana. Global health action. 2014;7:21292. Epub 2014/04/22. doi: 10.3402/gha.v7.21292. PubMed PMID: 24746141; PubMed Central PMCID: PMCPmc3991840.

94. Mitra S, Posarac A, Vick B. Disability and Poverty in Developing Countries: A Multidimensional Study. World Development. 2013;41:1-18. doi: 10.1016/j.worlddev.2012.05.024. PubMed PMID: WOS:000314016800001.

95. Mokhtari M, Dehghan SF, Asghari M, Ghasembaklo U, Mohamadyari G, Azadmanesh SA, et al. Epidemiology of mental health problems in female students: a questionnaire survey. Journal of epidemiology and global health. 2013;3(2):83-8. Epub 2013/07/17. doi: 10.1016/j.jegh.2013.02.005. PubMed PMID: 23856569.

96. Mont D, Cuong NV. Disability and poverty in Vietnam. The World Bank Economic Review. 2011;25(2):323-59.

97. Myer L, Stein DJ, Grimsrud A, Seedat S, Williams DR. Social determinants of psychological distress in a nationally-representative sample of South African adults. Social science & medicine (1982). 2008;66(8):1828-40. Epub 2008/02/27. doi: 10.1016/j.socscimed.2008.01.025. PubMed PMID: 18299167; PubMed Central PMCID: PMCPMC3203636.

98. Nakua EK, Otupiri E, Dzomeku VM, Owusu-Dabo E, Agyei-Baffour P, Yawson AE, et al. Gender disparities of chronic musculoskeletal disorder burden in the elderly Ghanaian population: study on global ageing and adult health (SAGE WAVE 1). BMC Musculoskeletal Disorders. 2015;16(204). PubMed PMID: 20153318374.

99. Natale JE, Joseph JG, Bergen R, Thulasiraj RD, Rahmathullah L. PREVALENCE OF CHILDHOOD DISABILITY IN A SOUTHERN INDIAN CITY - INDEPENDENT EFFECT OF SMALL DIFFERENCES IN SOCIAL-STATUS. International Journal of Epidemiology. 1992;21(2):367-72. doi: 10.1093/ije/21.2.367. PubMed PMID: WOS:A1992JB20600025.

100. Nguyen AJ, Haroz EE, Mendelson T, Bass J. Symptom Endorsement and Sociodemographic Correlates of Postnatal Distress in Three Low Income Countries. Depression Research & Treatment. 2016:1-11. doi: 10.1155/2016/1823836. PubMed PMID: 112982041.

101. Nguyen TT, Tran TD, Tran T, La B, Nguyen H, Fisher J. Postpartum change in common mental disorders among rural Vietnamese women: incidence, recovery and risk and protective factors. The British journal of psychiatry : the journal of mental science. 2015;206(2):110-5. Epub 2014/11/15. doi: 10.1192/bjp.bp.114.149138. PubMed PMID: 25395687.

102. Norris FH, Murphy AD, Baker CK, Perilla JL, Rodriguez FG, Rodriguez Jde J. Epidemiology of trauma and posttraumatic stress disorder in Mexico. Journal of abnormal psychology. 2003;112(4):646-56. Epub 2003/12/17. doi: 10.1037/0021-843x.112.4.646. PubMed PMID: 14674876.

103. Ou JJ, Shi LJ, Xun GL, Chen C, Wu RR, Luo XR, et al. Employment and financial burden of families with preschool children diagnosed with autism spectrum disorders in urban China: results from a descriptive study. BMC Psychiatry. 2015;15:3. Epub 2015/01/23. doi: 10.1186/s12888-015-0382-4. PubMed PMID: 25608486; PubMed Central PMCID: PMCPmc4307683.

104. Palmer MG. Inequalities in universal health coverage: evidence from Vietnam. World Development. 2014;64:384-94. PubMed PMID: 20143352868.

105. Palmer MG, Nguyen Thi Minh T, Quach Thi Ngoc Q, Dang Sy D, Hoang Van H, Berry HL. Disability measures as an indicator of poverty: A case study from Viet Nam. Journal of International Development. 2012;24:S53-S68. doi: 10.1002/jid.1715. PubMed PMID: WOS:000298881000004.

106. Patel V, Kirkwood BR, Pednekar S, Weiss H, Mabey D. Risk factors for common mental disorders in women - Population-based longitudinal study. British Journal of Psychiatry. 2006;189:547-55. doi: 10.1192/bjp.bp.106.022558. PubMed PMID: WOS:000242680200012.

107. Peres MA, Bastos JL, Watt RG, Xavier AJ, Barbato PR, D'Orsi E. Tooth loss is associated with severe cognitive impairment among older people: findings from a population-based study in Brazil. Aging & Mental Health. 2015;19(10):876-84. doi: 10.1080/13607863.2014.977770. PubMed PMID: WOS:000357325400002.

108. Petresco S, Anselmi L, Santos I, Barros A, Fleitlich-Bilyk B, Barros F, et al. Prevalence and comorbidity of psychiatric disorders among 6-year-old children: 2004 Pelotas Birth Cohort. Social Psychiatry & Psychiatric Epidemiology. 2014;49(6):975-83. doi: 10.1007/s00127-014-0826-z. PubMed PMID: 96107639.

109. Pham HL, Kizuki M, Takano T, Seino K, Watanabe M. Out-of-pocket Costs of Disabilities and Their Association with Household Socioeconomic Status Among School-aged Children in Vietnam. Journal of Rural Medicine. 2013;8(2):212-21. PubMed PMID: 25649134; PubMed Central PMCID: PMCPMC4309337.

110. Pheula GF, Rohde LA, Schmitz M. Are family variables associated with ADHD, inattentive type? A case―control study in schools. European child & adolescent psychiatry. 2011;20(3):137-45. PubMed PMID: 894620690; 23952262.

111. Ploubidis GB, Mathenge W, Stavola Bd, Grundy E, Foster A, Kuper H. Socioeconomic position and later life prevalence of hypertension, diabetes and visual impairment in Nakuru, Kenya. (Special Issue: The life course: Challenges and opportunities for public health research.). International Journal of Public Health. 2013;58(1):133-41. PubMed PMID: 20133064969.

112. Quadros LdCMd, Quevedo LdA, Motta JVdS, Carraro A, Ribeiro FG, Horta BL, et al. Social Mobility and Mental Disorders at 30 Years of Age in Participants of the 1982 Cohort, Pelotas, Rio Grande Do Sul – RS. PLoS ONE. 2015;10(10):1-11. doi: 10.1371/journal.pone.0136886. PubMed PMID: 110229748.

113. Rajkumar AP, Thangodurai P, Senthilkumar P, Goyathri K, Prince M, Jacob KS. Nature, prevalence and factors associated with depression among the elderly in a rural south Indian community. International Psychogeriatrics. 2009;21(2):372-8. doi: 10.1017/s1041610209008527. PubMed PMID: WOS:000264911000020.

114. Razzaque A, Nahar L, Khanam MA, Streatfield PK. Socio-demographic differentials of adult health indicators in Matlab, Bangladesh: self-rated health, health state, quality of life and disability level. Global Health Action. 2010;3:70-7. doi: 10.3402/gha.v3i0.4618. PubMed PMID: WOS:000208160500010.

115. Rischewski D, Kuper H, Atijosan O, Simms V, Jofret-Bonet M, Foster A, et al. Poverty and musculoskeletal impairment in Rwanda. Transactions of the Royal Society of Tropical Medicine & Hygiene. 2008;102(6):608-17. PubMed PMID: 18430444.

116. Rocha SV, de Almeida MM, de Araujo TM, Virtuoso JS, Jr. Prevalence of common mental disorders among the residents of urban areas in Feira de Santana, Bahia. Revista brasileira de epidemiologia = Brazilian journal of epidemiology. 2010;13(4):630-40. Epub 2010/12/25. PubMed PMID: 21180852.

117. Saha SK, Sanyal D, Bhattacharyya A, Bhattacharyya R, Barman N, Mukherjee A. A study on cognitive status of 50 years and above aged non-demented women in a rural area of West Bengal. Journal of the Indian Medical Association. 2010;108(11):726-9. PubMed PMID: 21510566.

118. Santos IS, Matijasevich A, Barros AJD, Barros FC. Antenatal and postnatal maternal mood symptoms and psychiatric disorders in pre-school children from the 2004 Pelotas Birth Cohort. Journal of Affective Disorders. 2014;164:112-7. doi: 10.1016/j.jad.2014.04.033. PubMed PMID: 96186313.

119. Scazufca M, Menezes PR, Araya R, Rienzo VDd, Almeida OP, Gunnell D, et al. Risk factors across the life course and dementia in a Brazilian population: results from the Sao Paulo Ageing & Health Study (SPAH). International Journal of Epidemiology. 2008;37(4):879-90. PubMed PMID: 20083276104.

120. Sengupta P, Benjamin AI. Prevalence of depression and associated risk factors among the elderly in urban and rural field practice areas of a tertiary care institution in Ludhiana. Indian Journal of Public Health. 2015;59(1):3-8. PubMed PMID: 20153115405.

121. Sengupta P, Benjamin AI, Yashpal S, Ashoo G. Prevalence and correlates of cognitive impairment in a north Indian elderly population. WHO South East Asia Journal of Public Health. 2014;3(2):135-43. PubMed PMID: 20153244514.

122. Shams G, Foroughi E, Esmaili Y, Amini H, Ebrahimkhani N. Prevalence rates of obsessive-compulsive symptoms and psychiatric comorbidity among adolescents in Iran. Acta Medica Iranica. 2011;49(10):680-7. PubMed PMID: 20113377698.

123. Sharifi V, Amin-Esmaeili M, Hajebi A, Motevalian A, Radgoodarzi R, Hefazi M, et al. Twelve-month prevalence and correlates of psychiatric disorders in Iran: the Iranian Mental Health Survey, 2011. Archives of Iranian medicine. 2015;18(2):76-84. Epub 2015/02/04. doi: 015182/aim.004. PubMed PMID: 25644794.

124. Singh RB, Singh Rao R, Thakur AS, Srivastav S, Niaz MA, Shinde SN. Prevalence and risk factors of cognitive deficits and dementia in relation to socioeconomic class in an elderly population of India. Journal of Anti-Aging Medicine. 1999;2(2):141-7. PubMed PMID: 1999318290.

125. Soares WB, Ribeiz SRI, Bassitt D, de Oliveira MC, Bottino CMC. Psychotic symptoms in older people without dementia from a brazilian community-based sample. International journal of Geriatric Psychiatry. 2015;30:437-45. PubMed PMID: 71629061.

126. Sosa AL, Albanese E, Stephan BCM, Dewey M, Acosta D, Ferri CP, et al. Prevalence, Distribution, and Impact of Mild Cognitive Impairment in Latin America, China, and India: A 10/66 Population-Based Study. Plos Medicine. 2012;9(2). doi: 10.1371/journal.pmed.1001170. PubMed PMID: WOS:000301222600003.

127. Sozmen K, Unal B. Socioeconomic Inequalities in non-communicable diseases and self assessed health in Turkey. Iranian Journal of Public Health. 2014;43(6):736-48. PubMed PMID: 20143247544.

128. Subbaraman R, Nolan L, Shitole T, Kiran S, Shrutika S, Sood K, et al. The psychological toll of slum living in Mumbai, India: a mixed methods study. Social Science & Medicine. 2014;119:155-69. PubMed PMID: 20143411372.

129. Taha AA, Pratt SR, Farahat TM, Abdel-Rasoul GM, Albtanony MA, Elrashiedy A-LE, et al. Prevalence and Risk Factors of Hearing Impairment Among Primary-School Children in Shebin El-Kom District, Egypt. American Journal of Audiology. 2010;19(1):46-60. doi: 10.1044/1059-0889(2010/09-0030. PubMed PMID: WOS:000289434900007.

130. Topuzoglu A, Binbay T, Ulas H, Elbi H, Tanik FA, Zagli N, et al. The epidemiology of major depressive disorder and subthreshold depression in Izmir, Turkey: Prevalence, socioeconomic differences, impairment and help-seeking. Journal of affective disorders. 2015;181:78-86. Epub 2015/05/02. doi: 10.1016/j.jad.2015.04.017. PubMed PMID: 25933098.

131. Trani JF, Bakhshi P, Kuhlberg J, Narayanan SS, Venkataraman H, Mishra NN, et al. Mental illness, poverty and stigma in India: a case-control study. BMJ open. 2015a;5(2):e006355. Epub 2015/02/26. doi: 10.1136/bmjopen-2014-006355. PubMed PMID: 25712818; PubMed Central PMCID: PMCPmc4342591.

132. Trani J-F, Bakhshi P, Myers Tlapek S, Lopez D, Gall F. Disability and Poverty in Morocco and Tunisia: A Multidimensional Approach. Journal of Human Development & Capabilities. 2015b;16(4):518-48. doi: 10.1080/19452829.2015.1091808. PubMed PMID: 112064860.

133. Trani J-F, Biggeri M, Mauro V. The Multidimensionality of Child Poverty: Evidence from Afghanistan. Social Indicators Research. 2013;112(2):391-416. doi: 10.1007/s11205-013-0254-6 .

http://dx.doi.org/10.1007/s11205-013-0253-7. PubMed PMID: 1463214800; 17984956.

134. Trani J-F, Loeb M. Poverty and disability: A vicious circle? Evidence from Afghanistan and Zambia. Journal of International Development. 2012;24:S19-S52. doi: 10.1002/jid.1709. PubMed PMID: WOS:000298881000003.

135. Vukovic D, Bjegovic V, Vukovic G. Prevalence of chronic diseases according to socioeconomic status measured by wealth index: health survey in Serbia. Croatian Medical Journal. 2008;49(6):832-41. PubMed PMID: 19090609; PubMed Central PMCID: PMCPMC2621032.

136. Wandera SO, Ntozi J, Kwagala B. Prevalence and correlates of disability among older Ugandans: evidence from the Uganda National Household Survey. Global Health Action. 2014;7(25686). PubMed PMID: 20153045020.

137. Wang H, Xiaozhao YY, Tingzhong Y, Cottrell RR, Lingwei Y, Xueying F, et al. Socioeconomic inequalities and mental stress in individual and regional level: a twenty one cities study in China. International Journal for Equity in Health. 2015a;14(1):1-7. doi: 10.1186/s12939-015-0152-4. PubMed PMID: 102766826.

138. Wang L, Feng Z, Yang G, Yang Y, Dai Q, Hu C, et al. The epidemiological characteristics of depressive symptoms in the left-behind children and adolescents of Chongqing in China. Journal of Affective Disorders. 2015b;177:36-41. PubMed PMID: 20153158602.

139. Wang S, Kou C, Liu Y, Li B, Tao Y, D'Arcy C, et al. Rural-urban differences in the prevalence of chronic disease in northeast China. Asia-Pacific journal of public health / Asia-Pacific Academic Consortium for Public Health. 2015c;27(4):394-406. Epub 2014/09/24. doi: 10.1177/1010539514551200. PubMed PMID: 25246500.

140. Wang ZJ, Du W, Pang L, Zhang L, Chen G, Zheng X. Wealth inequality and mental disability among the Chinese population: a population based study. International Journal of Environmental Research and Public Health. 2015d;12(10):13104-17. PubMed PMID: 20153407900.

141. Weobong B, Soremekun S, Ten Asbroek AH, Amenga-Etego S, Danso S, Owusu-Agyei S, et al. Prevalence and determinants of antenatal depression among pregnant women in a predominantly rural population in Ghana: The DON population-based study (English). Journal of affective disorders. 2014;165:1-7.

142. Williams JS, Ng N, Peltzer K, Yawson A, Biritwum R, Maximova T, et al. Risk factors and disability associated with low back pain in older adults in low- and middle-income countries. Results from the WHO study on global AGEing and adult health (SAGE). PLoS ONE. 2015;10 (6) (no pagination)(e0127880). PubMed PMID: 2015151788.

143. Wu F, Guo Y, Kowal P, Jiang Y, Yu M, Li X, et al. Prevalence of Major Chronic Conditions among Older Chinese Adults: The Study on Global AGEing and Adult Health (SAGE) Wave 1. PLoS ONE. 2013;8(9). PubMed PMID: 2013587708.

144. Wu Z, Xu J, He L. Psychological consequences and associated risk factors among adult survivors of the 2008 Wenchuan earthquake. BMC Psychiatry. 2014;14:126. Epub 2014/05/02. doi: 10.1186/1471-244x-14-126. PubMed PMID: 24779914; PubMed Central PMCID: PMCPmc4013305.

145. Xavier Gomez-Olive F, Thorogood M, Clark BD, Kahn K, Tollman SM. Assessing health and well-being among older people in rural South Africa. Global Health Action. 2010;3(Suppl 2):23-35.

146. Xiang Y-T, XinMa, Cai Z-J, Li S-R, Xiang Y-Q, Guo H-L, et al. Prevalence and socio-demographic correlates of schizophrenia in Beijing, China. Schizophrenia research. 2008;102(1-3):270-7. PubMed PMID: 61936440; 20860061.

147. Xie ZH, Bo SY, Zhang XT, Liu M, Zhang ZX, Yang XL, et al. Sampling survey on intellectual disability in 0 similar to 6-year-old children in China. Journal of Intellectual Disability Research. 2008;52:1029-38. doi: 10.1111/j.1365-2788.2008.01048.x. PubMed PMID: WOS:000261064800002.

148. Zainal M, Masran L, Ropilah AR. Blindness and visual impairment amongst rural Malays in Kuala Selangor, Selangor. Medical Journal of Malaysia. 1998;53(1):46-50. PubMed PMID: 10968137.

149. Zheng X, Chen R, Li N, Du W, Pei L, Zhang J, et al. Socioeconomic status and children with intellectual disability in China. Journal of Intellectual Disability Research. 2012;56(2):212-20. PubMed PMID: 21917049.

150. Zhou X, Bi B, Zheng L, Li Z, Yang H, Song H, et al. The prevalence and risk factors for depression symptoms in a rural Chinese sample population. PLoS ONE. 2014;9(6). PubMed PMID: 20143289810.
